# Resting-state global brain activity affects early β-amyloid accumulation in default mode network

**DOI:** 10.1038/s41467-023-43627-y

**Published:** 2023-11-27

**Authors:** Feng Han, Xufu Liu, Richard B. Mailman, Xuemei Huang, Xiao Liu

**Affiliations:** 1 Department of Biomedical Engineering, The Pennsylvania State University, State College, PA USA; 2 Departments of Neurology and Pharmacology, Translational Brain Research Center, Pennsylvania State University College of Medicine and Milton S. Hershey Medical Center, Hershey, PA USA; 3 Departments of Radiology, Neurosurgery, and Kinesiology, Translational Brain Research Center, Pennsylvania State University and Milton S. Hershey Medical Center, Hershey, PA USA; 4 Institute for Computational and Data Sciences, The Pennsylvania State University, State College, PA USA

**Keywords:** Alzheimer's disease, Alzheimer's disease, Neural circuits

## Abstract

It remains unclear why β-amyloid (Aβ) plaque, a hallmark pathology of Alzheimer’s disease (AD), first accumulates cortically in the default mode network (DMN), years before AD diagnosis. Resting-state low-frequency ( < 0.1 Hz) global brain activity recently was linked to AD, presumably due to its role in glymphatic clearance. Here we show that the preferential Aβ accumulation in the DMN at the early stage of Aβ pathology was associated with the preferential reduction of global brain activity in the same regions. This can be partly explained by its failure to reach these regions as propagating waves. Together, these findings highlight the important role of resting-state global brain activity in early preferential Aβ deposition in the DMN.

## Abstract (web summary)

Why β-amyloid plaque initially accumulates in the default mode network of the cortex before Alzheimer’s disease diagnosis is not known. Here, the authors show that this accumulation is associated with a reduction of global brain activity in these regions.

## Introduction

Biomarkers of Alzheimer’s disease (AD) become abnormal sequentially^1–4^. Among them, decreased Aβ42 in cerebrospinal fluid (CSF) and increased Aβ accumulation in the brain are the earliest, occurring decades before clinical diagnosis of dementia^1,5^. As AD progresses, cortical Aβ accumulation follows a specific spatial trajectory. It spreads from areas predominantly in the default mode network (DMN), including precuneus, posterior cingulate, and orbitofrontal cortices, to a set of lower-order sensory-motor areas, including precentral, postcentral, pericalcarine, and lingual regions^5^. It is, however, unclear why cortical Aβ accumulation appears to start in regions of the DMN. Current hypotheses have focused on the production side and attributed the vulnerability of the DMN to its high neuronal activity^6–8^ and/or metabolic stress^6,9,10^. Consistent with these hypotheses, Aβ secretion and deposition increase in proportion to neuronal activity^7,8,11,12^.

The process of Aβ accumulation could be impacted equally by clearance. The clearance aspect received little attention until recent evidence suggested its role in AD^13,14^. In particular, the glymphatic system, through its key functional role in clearing brain “waste” through CSF flow via the perivascular pathway, may contribute markedly to whether Aβ accumulates and affects AD progression^13,15,16^. Glymphatic clearance was linked recently to spontaneous low-frequency ( < 0.1 Hz) brain-wide brain activity assessed via the global blood oxygenation level-dependent (gBOLD) signal in resting-state functional MRI (rsfMRI). This gBOLD is greater during sleep and also coupled to CSF movement^17,18^, similar to glymphatic function. The gBOLD-CSF coupling thus has been proposed to reflect glymphatic function and, indeed has been associated with AD pathology as well as Parkinson’s disease (PD) cognitive impairment^17,19^.

Like Aβ accumulation, the impairment of glymphatic clearance in neurodegenerative diseases is unlikely to be temporally and spatially uniform. Temporally, glymphatic function measured by gBOLD-CSF coupling gradually decreases as AD progresses, in parallel with decreases in cognitive function^17^. Similarly, the coupling does not decrease in PD until cognitive decline is detectable^19^. Recent data on glymphatic measures suggests that there may be some degree of spatial heterogeneity of glymphatic function^20–22^. For example, patients with idiopathic intracranial hypertension often report cognitive impairment and also have lower glymphatic function, especially in brain regions (e.g., frontal, temporal, and cingulate cortices) where there is early deposition of Aβ and tau in AD^23^. Together, this generated the hypothesis that the spreading of protein aggregates in AD follows the direction of glymphatic inflow^14^. This hypothesis, however, assumes no role for neural activity, and is inconsistent with evidence supporting the involvement of neural pathways in Aβ spreading^24–26^.

The link between glymphatic clearance and resting-state global brain activity^17^ may reconcile this apparent paradox, suggesting the hypothesis that neural activity may be driving glymphatic function and, ultimately, affecting the spreading pattern of toxic proteins. Consistent with this hypothesis, resting-state global brain activity, measured either by fMRI (i.e., gBOLD) or electrophysiology, has shown a sensory-dominant (i.e., much stronger in the sensory-motor areas) pattern^27–29^ that is opposite to the spatial distribution of early Aβ deposition^5^. This global activity recently was found often to take the form of propagating waves between the DMN and the sensory-motor areas^30,31^, resembling the spreading trajectory of Aβ in AD. This spatiotemporal correspondence leads to the question of whether, and how, the resting-state global brain activity and its coupling to CSF flow, affect the preferential Aβ deposition in the DMN in early AD. Given its potential effect on connectivity assessments^32,33^, it also raises the question of whether global brain activity contributes to Aβ-associated changes in functional connectivity in the DMN^5,34^. Using the data from the Alzheimer’s Disease Neuroimaging Initiative (ADNI), we now address these questions by investigating, spatially and temporally, the potential link of global brain activity (measured by gBOLD) and its coupling to CSF inflow to the aggregation of Aβ^5^.

## Results

### Demographics and clinical states of the study subjects

**Fig. 1:**
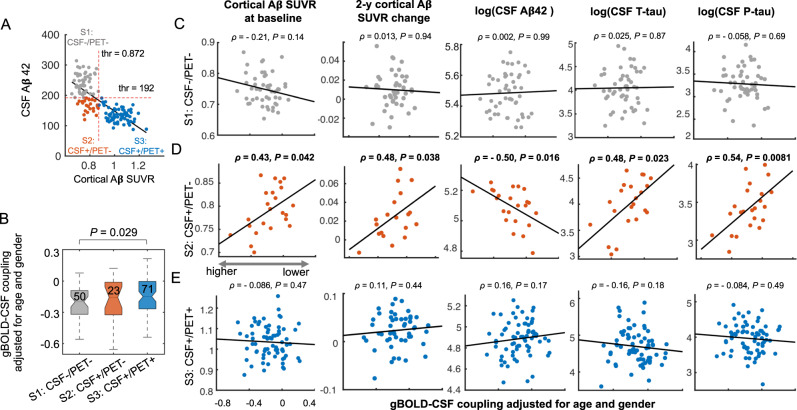
Stage-dependent associations between various markers of protein aggregation and the global glymphatic function quantified by the gBOLD-CSF (global BOLD–cerebrospinal fluid) coupling. **A** The whole cohort of 144 subjects were categorized into three stages based on the level of CSF amyloid-beta 42 (Aβ42) (CSF+: <192 ng/L) and cortical Aβ standardized uptake value ratio (SUVR) (PET+: cortical Aβ > 0.872 SUVR) following the same procedure used in a previous study^5^. **B** The gBOLD-CSF coupling strength decreased gradually from S1 to S3 as the Aβ pathology progresses (*P* = 0.044; ordinal regression, two-sided), and the significant (*P* = 0.029, two-sample *t*-test, two-sided) group difference was found between S1 and S3. The gBOLD-CSF coupling was computed from resting-state fMRI data (see details in Methods and Figure S1; age and gender adjusted) to quantify the global glymphatic function. The bottom and top edges and the central line of the boxes represent the first and third quartiles and the median respectively, whereas the whiskers represent the minimum and maximum. The “notches” on the boxes delineate the 95% conference interval for the median. The number of subjects in each subgroup is shown on the boxes. **C**–**E** Associations between the gBOLD-CSF coupling and various markers of protein aggregation in different Aβ stages. In S2 (CSF+/PET−), the lower glymphatic function (i.e., less negative gBOLD-CSF coupling) was strongly associated (Spearman’s correlation) with the higher cortical Aβ SUVR (*ρ* = 0.43, *P* = 0.042; *N* = 23; *P*_*FDR-corrected*_ = 0.13, False Discovery Rate (FDR) applied for each column), its larger increase in the following 2 years (*ρ* = 0.48, *P* = 0.038, *P*_*FDR-corrected*_ = 0.11; *N* = 19), less Aβ42 (natural logarithm; *ρ* = − 0.50, *P* = 0.016, *P*_*FDR-corrected*_ = 0.048; *N* = 23), but higher T-tau (*ρ* = 0.48, *P* = 0.023, *P*_*FDR-corrected*_ = 0.069; *N* = 22*)* and P-tau level (*ρ* = 0.54, *P* = 0.0081, *P*_*FDR-corrected*_ = 0.024; *N* = 23*)* in CSF. Each dot represents one subject. Source data are provided as a Source Data file. The linear regression line was estimated based on the linear least-squares fitting (the same hereinafter unless noted otherwise).

We analyzed rsfMRI and [^18^F]florbetapir positron emission tomography (PET) data from 144 participants (72.6 ± 7.5 years; 73 females) of the ADNI project^35^. The participants included 28 healthy controls, 21 subjects with significant memory concern (SMC), 72 with mild cognitive impairment (MCI), and 23 AD patients (see Table 1 for details). The cohort was selected based on the availability of rsfMRI and PET measurements of Aβ ([^18^F]florbetapir). Following a published procedure^5^, the participants were sub-grouped into three stages based on their cortical Aβ Standardized Uptake Value Ratio (SUVR; referring to the composite region, see Methods) from PET (i.e., PET+ if cortical Aβ > 0.872 SUVR) and CSF Aβ42 (i.e., CSF+ if <192 ng/L). The stages (Fig. 1A) were: non-Aβ-accumulators (S1: CSF-/PET-); early-Aβ-accumulators (S2: CSF+/PET−); and late-Aβ-accumulators (S3: CSF+/PET+). No significant age and gender differences were found among the stages, except for the gender ratio between S1 and S2 (*P* = 0.049, Fisher exact test, uncorrected). A subset of 112 participants also underwent Aβ-PET scans approximately two years later, the data from which were used for computing two-year cortical Aβ changes.

**Table 1:** Participant characteristics

	Stage 1 (S1)	Stage 2 (S2)	Stage 3 (S3)	*P*-value		
*N* = 144	CSF-/PET- (*N* = 50)	CSF+/PET- (*N* = 23)	CSF+/PET+(*N* = 71)	S2 vs S1	S3 vs S1	S3 vs S2
Age	71.7 (8.0)	70.5 (7.3)	73.9 (7.0)	0.54	0.12	0.051
Gender (M/F)	22/28	16/7	33/38	**0.049**	0.85	0.060
Group (AD:MCI:SMC:Control)	2:23:12:13	0:12:3:8	21:37:6:7	–	–	–
APOE4# (0:1:2)	42:8:0	15:5:3	19:38:14	–	–	–

Data represent the mean and standard deviation (in parentheses) unless otherwise indicated. Pairwise comparisons were performed based on the two-sample *t* test (two-sided) for all measures except for gender where a Fisher exact test was used (significant result is highlighted with bold formatting).

*M/F* male/female, *AD* Alzheimer’s disease group, *MCI* mild cognitive impairment, *SMC* significant memory concern, *APOE4#* the number of APOE ε4 carrying, *CSF*+ <192 ng/L, *PET* *+*  cortical Aβ > 0.872 SUVR.

### FMRI-based glymphatic measure is strongly associated with protein aggregation in the early stage of Aβ pathology

The gBOLD-CSF coupling^17^ (e.g., Fig. S1) that is of potential relevance to glymphatic function is lower as Aβ pathology increases with advancing stages (i.e., from S1 to S3; *P* = 0.044, ordinal regression; Fig. 1B). This is similar to its modulation across four different clinical states (i.e., control to SMC to MCI to AD) shown previously^17^. The associations between gBOLD-CSF coupling and various markers of protein aggregation then were calculated for each of the three stages of Aβ pathology (Fig. 1C–E). The gBOLD-CSF coupling strength was correlated strongly with protein aggregation, but only in the early-Aβ-accumulators in whom CSF Aβ42 was significantly lower but cortical Aβ had just begun to accumulate^5^. Within this early stage of Aβ pathology (S2), the subjects with a lower gBOLD-CSF coupling had higher cortical Aβ SUVR (Spearman’s *ρ* = 0.43, *P* = 0.042), more Aβ accumulations in the subsequent two years (*ρ* = 0.48, *P* = 0.038), lower CSF Aβ42 (*ρ* = − 0.50, *P* = 0.016), and higher CSF total and phosphorylated tau (T-tau: *ρ* = 0.48, *P* = 0.023; P-tau: *ρ* = 0.54, *P* = 0.0081; Fig. 1D). These associations between protein markers and gBOLD-CSF coupling remained strong after adjusting for the baseline cortical Aβ (Fig. S2), supporting the critical role of early-Aβ-accumulators on which our subsequent analyses focused.

### Preferential Aβ accumulation in the DMN is related to reduced local BOLD-CSF coupling in the early stage of Aβ pathology

**Fig. 2:**
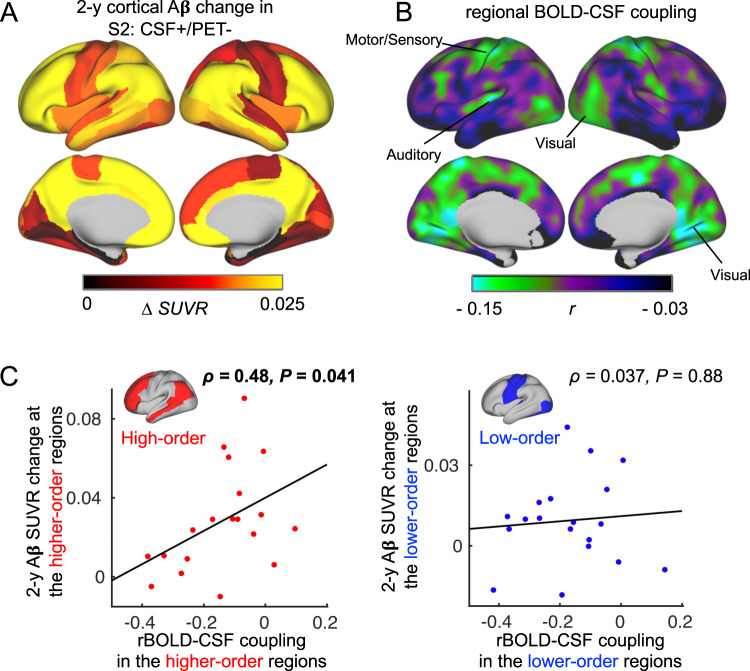
The rBOLD-CSF (regional BOLD-cerebrospinal fluid) coupling was associated with the amyloid-beta (Aβ) accumulation locally at the extended default mode network (DMN) areas among the early Aβ accumulators (CSF+/PET-). **A** The two-year (2-y) Aβ accumulation averaged over 19 early accumulators with the longitudinal data showed much higher values in the extended DMN regions, including the DMN and frontoparietal network (FPN). **B** The averaged map (*N* = 19) of the rBOLD-CSF coupling was less negative in the DMN regions, suggestive of weaker glymphatic function. **C** The association between the rBOLD-CSF coupling (adjusted for age and gender) and the 2-y Aβ SUVR change is significant (Spearman’s *ρ* = 0.48, *P* = 0.041, two-sided, *P*_*FDR-corrected*_ = 0.082; *N* = 19) in the higher-order brain networks (left), but not so (*ρ* = 0.037, *P* = 0.88, *P*_*FDR-corrected*_ = 0.88) in the lower-order sensory-motor regions (right). Each dot represents one CSF+/PET− subject. The higher-order area mask covers the DMN and FPN, whereas the lower-order mask includes the sensory, visual, and auditory networks (Figure S3). Source data are provided as a Source Data file. Parcel-based correlations between the two can be found in Fig. S5.

We then investigated how cortical Aβ accumulation, glymphatic function, and their association varied spatially across the cortex in early Aβ accumulators (CSF+/PET−). Consistent with a prior report^5^, the early accumulators had much higher Aβ accumulation in the subsequent two years in a set of brain networks related to higher-order functions [e.g., DMN and frontoparietal network (FPN)] than in the lower-order sensory-motor areas (Fig. 2A, see detailed results in Figs. S3 and S4). Since gBOLD is simply the mean of regional BOLD (rBOLD) signals, we used the coupling between rBOLD and CSF to quantify how local brain regions were differently involved in the CSF flow of potentially glymphatic relevance. The rBOLD-CSF coupling (Fig. 2B) displayed a spatial pattern opposite to the Aβ accumulation pattern, with the higher-order networks having weaker coupling (i.e., less negative). This rBOLD-CSF coupling was significantly (*ρ* = 0.48, *P* = 0.041) associated with the two-year Aβ change in the high-order brain networks (Fig. 2C, left), but not (*ρ* = 0.037, *P* = 0.88) the sensory-motor areas (Fig. 2C, right). This is consistent with parcel-based analysis showing the positive correlations between the two mostly in the higher-order brain regions (Fig. S5). It suggested that the higher-order brain networks, but not lower-order networks less prone to Aβ accumulation, were affected by the process that is quantified by BOLD-CSF coupling and potentially linked to the glymphatic function.

### Preferential Aβ accumulation in the DMN is related to locally reduced gBOLD presence in the early stage of Aβ pathology

**Fig. 3:**
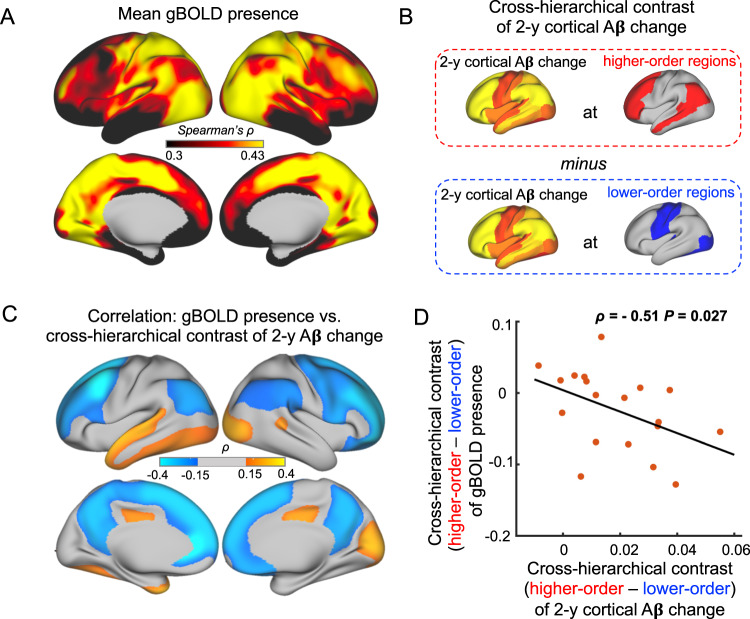
Preferential reduction of global BOLD (gBOLD) presence at the higher-order brain networks is correlated with the preferential amyloid-beta (Aβ) accumulation in the same areas. **A** The averaged map of gBOLD presence in the CSF+/PET− subjects (early accumulators) showed a sensory-dominant pattern similar (Spearman’s *ρ* = − 0.68, *P* = 0, two-sided) to the rBOLD-CSF (regional BOLD-cerebrospinal fluid) coupling map. **B** The cross-hierarchical contrast of the 2-y Aβ standardized uptake value ratio (SUVR) change was computed for each subject as the difference between the higher- and lower-order regions. This cross-hierarchy contrast quantifies the extent to which Aβ is preferentially accumulated in the higher-order regions as compared with the lower-order areas. **C** The gBOLD presence (adjusted for age and gender) at the higher-order brain regions showed the strongest negative correlations with the cross-hierarchical contrast of 2-y Aβ change, suggesting the reduced gBOLD presence in these areas contributes to the preferential Aβ accumulation in the same regions in the following 2 years. **D** The cross-hierarchical contrast of the gBOLD presence (adjusted for age and gender) is significantly correlated (*ρ* = − 0.51, *P* = 0.027; two-sided, *N* = 19) with that of the 2-year cortical Aβ changes. Each dot represents one CSF+/PET− subject. Source data are provided as a Source Data file.

Given that the same CSF signal was used for a given participant, any spatial variation in the rBOLD-CSF (Fig. 2B) can be solely attributed to changes in rBOLD, presumably the contribution from gBOLD. Consistent with this notion, gBOLD peaks and their underlying neural signals have been shown to display a similar sensory-dominant pattern^27–29^. We thus quantified the gBOLD presence across the cortex by correlating rBOLD with gBOLD^29^. The resulting gBOLD presence map (Fig. 3A) displayed a sensory-dominant pattern similar to the rBOLD-CSF coupling, but opposite to Aβ accumulation.

To test if there was a tight link between the gBOLD presence and Aβ accumulation beyond the spatial similarity, we examined the cross-subject correlations between the two variables. We computed a cross-hierarchy contrast of the two-year Aβ accumulation for each subject (Fig. 3B). This quantifies the “preferential” Aβ accumulation in the higher-order regions as compared to the lower-order areas. The gBOLD presence at the higher-order regions was correlated negatively with this contrast (Fig. 3C), suggesting that the lower gBOLD presence was followed by preferential Aβ accumulation in these regions two years after baseline. The cross-hierarchical contrast also was computed for gBOLD presence, and was negatively correlated (*ρ* = −0.51, *P* = 0.027) with the cross-hierarchical contrast of two-year Aβ accumulation (Fig. 3D). Together, these data show that those early-Aβ-accumulators having greater preferential gBOLD reduction in higher-order networks also had a more preferential Aβ accumulation in the same regions in the subsequent two years.

### Lower gBOLD signal accounts for Aβ-associated hypoconnectivity in higher-order regions in the early stage of Aβ pathology

**Fig. 4:**
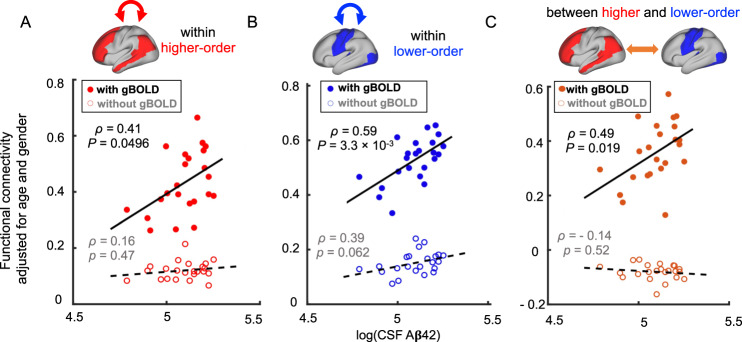
Associations between the functional connectivity and cerebrospinal fluid (CSF) amyloid-beta 42 (Aβ42) in the early Aβ accumulators (CSF+/PET−) were diminished by removing the global BOLD (gBOLD) component. The functional connectivity (adjusted for age and gender) within (**A**, **B**) and between (**C**) the higher- and lower-order regions is significantly correlated (Spearman’s; two-sided) with the CSF Aβ42 (natural logarithm) among the early Aβ accumulations (filled circles; *P*_*FDR-corrected*_ for the three panels were 0.0496, 0.0099, and 0.029, respectively). Re-assessing the connectivity after removing the gBOLD component led to these associations becoming non-significant for the connectivity related to the higher-order regions (**A** and **C**, open circles), and marginally significant for the connectivity within the lower-order region (**B**, circles). Each solid or hollow dot represents one CSF+/PET− subject. The regression slopes changed significantly (**A**: *P* = 0.0078; **B**: *P* < 0.0001; **C**: *P* = 0.0003) before and after the gBOLD removal in all cases. Source data are provided as a Source Data file.

Changes in gBOLD can affect functional connectivity (FC) measured by rBOLD correlations^32,33^. In a different cohort of early Aβ accumulators (CSF+/PET−) from the BioFINDER study^5^, the FC of the DMN was lower as CSF Aβ42 levels decreased. We thus asked whether the changes in gBOLD were responsible for the Aβ-associated connectivity changes. We first replicated the previous finding using the ADNI data. The FC within the higher-order DMN and FPN showed a significant correlation (*ρ* = 0.41, *P* = 0.0496) with the CSF Aβ42 in the early Aβ accumulators (CSF+/PET-) (Fig. 4A, filled circles). This association, however, was not unique to the FC of the higher-order brain networks. The FC within the sensory-motor networks (*ρ* = 0.59, *P* = 0.0033) and between the higher- and lower-order regions (*ρ* = 0.49, *P* = 0.019) also showed a significant correlation with the CSF Aβ42 level (Fig. 4B, C, filled circles). These less specific FC changes with CSF Aβ42 could be related to gBOLD. To test this hypothesis, FC was re-computed after regressing out gBOLD from rBOLD signals. The “gBOLD-removed” FC involving the higher-order regions did not then correlate with CSF Aβ42 (Fig. 4A, C, open circles), and the FC within the lower-order regions was only marginally correlated (*ρ* = 0.39, *P* = 0.062; Fig. 4B, open circles). All three CSF Aβ42-FC associations were significantly lower (*p* < 0.0078; slope comparison test) after the removal of gBOLD. These results suggest that gBOLD is related to Aβ-associated FC changes, and also that low gBOLD accounts for the hypoconnectivity associated with the low level of CSF Aβ42.

### Disengagement of gBOLD propagating waves from the DMN

**Fig. 5:**
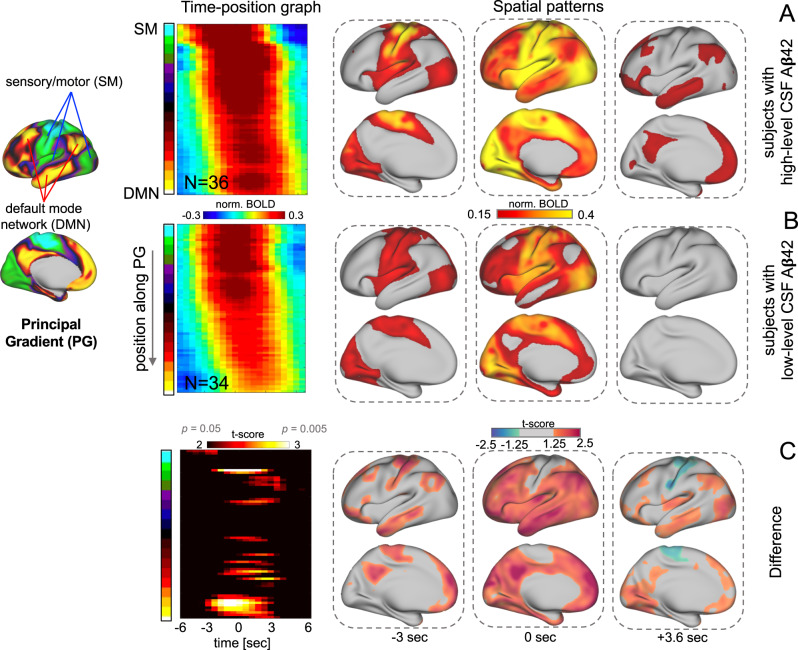
The global BOLD (gBOLD) is significantly different as the sensory-motor to default mode network (SM-to-DMN) propagating waves in the early amyloid (Aβ) accumulators with distinct cerebrospinal fluid (CSF) amyloid-beta 42 (Aβ42) levels. The gBOLD peaks showing propagations along the principal gradient (PG)^36^ (the left column) from the sensory-motor (SM) to DMN were identified following a previous study^30^. These gBOLD propagating waves were averaged within the early accumulators (*N* = 8 for each group) with the top 1/3 (**A**) and bottom 1/3 (**B**) CSF Aβ42 values. Their difference was shown in **C**. The average patterns of the SM-to-DMN propagations appear as the tilted bands in the time-position graph (2nd column) but more intuitive spatial patterns on the brain surface (3rd to 5th columns). The time-position graph segments (2nd column) are simply the mean of the detected SM-to-DMN propagation instances, and colors encode the mean values of the rBOLD signals. A two-sided (two-sample) *t*-test was used to compare the propagating waves from the sub-groups in the **A** and **B** (uncorrected). Source data are provided as a Source Data file.

Our final analysis was to understand the brain dynamics underlying the preferential gBOLD decrease in the higher-order brain networks. Global brain activity, measured either by gBOLD or global electrophysiology signal, can take the form of infra-slow propagating waves between the higher- and lower-order brain regions^30,31^, a direction described by the principal gradient (PG) of the functional brain connectivity (Fig. 5, left)^30,36^. Using a previously described method^30^, we identified and extracted gBOLD peaks showing propagations along PG directions. The gBOLD propagating waves were clear on the brain surface and manifested as tilted bands in the time-position graph along the PG direction (Fig. 5 and S6). We then compared the gBOLD propagations in two subgroups of the early accumulators with the highest- and lowest-third CSF Aβ42 values and distinct FC strength (Fig. 4; filled circles). Compared with the other group, the gBOLD propagations appear to be weaker in the low-level CSF Aβ42 group that has relative hypoconnectivity (Figs. 5 and S6). The difference is especially significant in the higher-order regions for the propagations from the sensory-motor regions to the DMN (Fig. 5B), suggesting this type of propagations largely failed to reach the DMN areas (Fig. 5C).

Of note, the major results presented above remain essentially the same after controlling for various factors, including head motion (Figs. S7 and S8), the number of discarded fMRI volumes (Fig. S9), the time lag between gBOLD and CSF signals (Fig. S10), the way of obtaining ROI parcels (Figure S11), the cognitively defined disease conditions (Fig. S12), the variability of fMRI parameters (Fig. S13), and pulse-pressure (Fig. S14).

## Discussion

We have demonstrated a link between resting-state global brain activity and the spatial and temporal features of Aβ accumulation in the early stage of Aβ pathology. Global glymphatic function, measured by the coupling between the global brain activity (gBOLD) and CSF flow, was strongly associated with various Aβ and tau markers at the early stage of Aβ pathology. The data support the critical role of glymphatic function at this transitional stage (CSF+/PET-) that features significantly lower CSF Aβ42, but early Aβ accumulation in higher order DMN regions in subsequent two years. In this early stage of Aβ pathology, the preferential Aβ deposition was preceded by reduced regional BOLD-CSF coupling that is attributed largely to the reduced gBOLD presence in the same regions. The reduced global brain activity contributed to hypoconnectivity, particularly in high-order brain networks, that are associated with the Aβ pathology. The disengagement of global brain activity from the DMN may be partly attributed to its failure to reach this higher-order network as propagating waves. Together, these results suggest that the resting-state global brain activity affects Aβ accumulation at the early stage of Aβ pathology and in a spatially differentiated way, presumably through its effect on glymphatic clearance.

Resting-state global brain activity has been linked to the cholinergic and memory systems, both of which are critically involved in AD. The gBOLD signal was initially found to be driven by global, but sensory-dominant, brain co-activations^27–29^, and more recently shown to propagate as waves along the gradient of cortical hierarchy^30,31^. This widespread brain co-activation was accompanied by specific de-activation in subcortical arousal-regulating areas, particularly the nucleus basalis at the basal forebrain and the locus coeruleus of the brainstem^28,30^. This has strong relevance to AD etiology since the cholinergic cell loss in these regions is a hallmark of AD^37,38^. Consistent with the involvement of arousal-regulating regions is a strong dependence of gBOLD on brain vigilance states^28,39,40^. In non-human primates, a causal relationship has been established between the deactivation of the basal forebrain cholinergic areas and the suppressed gBOLD^41^. At the single-neuron level, the resting-state global brain activity manifests as spiking cascades featuring sequential activation of the majority of neuronal populations^42^. The associated modulations of pupil size and delta-power (0.5–4 Hz) were similar to what has been observed for gBOLD^27,28,41,43^, confirming cholinergic involvement in these global brain dynamics. More importantly, the spiking cascade cycle strongly modulated the occurrence of hippocampal sharp-wave ripples known to be important for memory consolidation^44,45^, linking this global activity to the memory system^42^.

Resting-state global activity has been linked to AD through its role in Aβ clearance^13,46^. The clearance of brain waste by the glymphatic pathway^13,16^ involves CSF movement from the periarterial into the interstitial space, facilitated by astroglial aquaporin-4 (AQP4) channels. This flushes interstitial solutes including Aβ and tau^47^. The gBOLD initially was linked to glymphatic clearance due to its coupling to CSF movement and similar dependency on sleep^17,18^, and this coupling was then found to be correlated with various AD pathologies and also with cognitive decline in Parkinson’s disease (PD)^17,19^. There are several mechanisms by which the global activity may affect glymphatic clearance. Both the spiking cascade and gBOLD are accompanied by strong sympathetic changes, (e.g., pupil size^42,43,48^, cardiac and respiratory pulsations^49–53^, and heart rate variability^54^). Such phasic sympathetic activations may either constrict pial arteries directly^51,55^ to facilitate peri-arterial CSF movements, or achieve the same result indirectly by causing slow ( < 0.1 Hz) modulations of cardiac and respiratory pulsations that have been regarded as the major driving forces of glymphatic CSF flow^56,57^. Additionally, resting-state global activity also may modulate vascular tone beyond Virchow-Robin space via intrinsic subcortical vasoactive pathways^55^, particularly the basalocortical projections^58^, given its link to the cholinergic system. This is intriguing given the potential involvement of astrocytes. The perivascular nerves of these subcortical pathways abut primarily on astrocytic endfeet where most AQP4 is located, with a smaller proportion having direct contact with the vessel wall^55^. Global brain activity thus may facilitate glymphatic CSF flow via coordinated AQP4 activation. Consistent with this notion, gBOLD negative peaks have been found to be coupled to large astrocytic Ca^2+^ transients^59^. It is worth noting that the gBOLD and its coupling with CSF were intended to measure this global activity and associated processes of potentially glymphatic relevance, but not to directly measure the glymphatic clearance through the quantification of CSF flow.

Higher-order brain networks, particularly DMN, are known to be more vulnerable to early Aβ aggregation^5,60,61^. The association between the DMN BOLD and Aβ buildup has been explained from the production side and hypothetically attributed to high levels of brain activity and metabolism^6,9^, consistent with animal studies showing that increased brain activity leads to greater Aβ secretion and deposition^7,8,11,12,62^. It may not, however, explain why cognitive and social activities that recruit the higher-order brain regions reduce the risk of AD and dementia^63,64^. Moreover, glucose hypo-, but not hypermetabolism, was reported in preclinical stages of AD^65^, and also has been associated with the APOE ɛ4 heterogenicity of cognitively normal subjects^66^.

An alternative hypothesis has been posited recently to explain the Aβ spreading pattern from the clearance side^14^. It suggests that Aβ spreading roughly follows the direction of, and thus may be related to, glymphatic inflow. This notion, however, may be incompatible with significant evidence for the involvement of neural pathways in Aβ spreading^24–26^. For example, the regions of early Aβ accumulation regions resemble connectivity-defined networks (mostly DMN) more than early-perfused brain areas (Fig. 2A)^6,9^. Moreover, Aβ spreads preferentially between anatomically connected regions^6,7,25,67–69^. This apparent discrepancy may, however, be reconciled by considering the neural relevance of the glymphatic system to the resting-state global brain activity. The gBOLD is known to have a sensory-dominant pattern opposite to the early Aβ deposition^28^, and even to propagate as waves along a similar direction as Aβ spreading^30^. Here we went beyond just spatial correspondence between the resting-state brain activity and early, preferential Aβ accumulation and established a tight link, through cross-subject correlations, between lower gBOLD presence and Aβ accumulation. We showed that the early Aβ accumulators with a greater gBOLD reduction in DMN regions had higher Aβ accumulation in the same areas in the subsequent two years. This association appeared to be mediated by the locally reduced involvement in a global process of glymphatic relevance, as measured by the rBOLD-CSF coupling. In addition, the lower gBOLD presence may account for Aβ-associated changes in functional connectivity in AD^5,6,60,70^, and also may, in part, result from the changes of the global brain activity as propagating waves.

It is worth noting that all these findings greatly expand the previous finding of the relationship between the gBOLD-CSF coupling and AD pathology^17^ by linking the gBOLD-related process specifically to the preferential Aβ deposition in the DMN. Also, this coupling index was less sensitive to Aβ pathology at the late stage (S3: CSF+/PET+) but showed more specific and strong correlations with various protein markers at the early accumulators, suggesting the stage-dependent role of the gBOLD-related process in the glymphatic function. Given these important findings for the early accumulators, it should be noted the sample size for this group of CSF+/PET- subjects remains relatively small (23 subjects). This is due to the limited number of ADNI participants who were at the early stage of Aβ pathology and underwent multiple modalities of resting-state fMRI, longitudinal PET-Aβ, and CSF-Aβ. Nevertheless, these subjects with rapid Aβ changes are of great interest in Alzheimer’s research, and our data warrant future follow-up studies, with larger sample sizes and in independent cohorts, to validate and extend the findings.

In summary, our study provides preliminary evidence that the DMN disengagement from the global brain activity may explain, in part, its vulnerability to early Aβ accumulation. Future prospective studies are warranted to replicate these findings and may have a significant impact on the development of prevention strategies, in those at high risk of developing AD.

## Methods

### Participants and study data

We included 144 participants from the ADNI project (ADNI-GO and ADNI-2) according to the availability of rsfMRI, CSF Aβ42, and ^18^F-AV45 amyloid PET data. The present cohort consisted of healthy controls (*N* = 28), significant memory concern (SMC; *N* = 21) subjects, mild cognitive impairment (MCI; *N* = 72) subjects, and AD patients (*N* = 23), which were defined by ADNI (http://adni.loni.usc.edu/study-design/). We summarized the participant characteristics, including age, gender, and the number of APOE ε4 allele carrying. To investigate the longitudinal cortical Aβ accumulation, we identified and examined 112 participants, out of the 144, with 2-year follow-up (24.0±1.2 months) data of Aβ-PET. No participants in the present study experienced changes in the disease condition over the 2 years. All participants provided written informed consent. Investigators at each ADNI participating site obtained ethical approval from the individual institutional review board (IRB; http://adni.loni.usc.edu/wp-content/uploads/2013/09/DOD-ADNI-IRB-Approved-Final-protocol-08072012.pdf). ADNI data were collected per the principles of the Declaration of Helsinki (clinical trial registration number: NCT00106899 [ADNI] and NCT01231971 [ADNI2]).

The data of rsfMRI, Aβ-PET, Aβ42 level in CSF, total and phosphorylated tau at Thr181 in CSF (T-tau and P-tau), and APOE genotype at baseline were obtained from the same study visit (the visit codes were defined by ADNI; see details at https://adni.loni.usc.edu/wp-content/uploads/2008/07/inst_about_data.pdf). The files “UC Berkeley—AV45 Analysis [ADNI1, GO, 2, 3] (version: 2020-05-12)”^71,72^ and “APOE—Results [ADNI1, GO, 2, 3] (version: 2013-05-14)” summarized by ADNI were used to provide the Aβ-PET SUVR and APOE genotype data for the present study. CSF Aβ42, CSF T-tau, and CSF P-tau data for our cohort were obtained from the “UPENN CSF Biomarker Master [ADNI1, GO, 2] (version: 2016-07-05)”^73^. All the data above, as well as the rsfMRI data, are publicly accessible on the ADNI website (http://adni.loni.usc.edu/).

The use of de-identified data from the ADNI and the sharing of analysis results have been reviewed and approved by the Pennsylvania State University IRB (IRB#: STUDY00014669), and also strictly followed the ADNI data use agreements.

### Image acquisition and preprocessing

All rsfMRI were acquired at 3Tesla MR scanners from multiple ADNI participating sites following a unified protocol (http://adni.loni.usc.edu/methods/documents/mri-protocols/). The MRI data used in the current study was mostly collected in Philips scanners (Philips Medical Systems Philips, Amsterdam, Netherlands) with only one of 144 subjects’ data acquired by a Siemens MRI scanner (Siemens Medical Solutions, Siemens, Erlangen, Germany). Each imaging session included an MPRAGE sequence (echo time (TE) = 3.1 ms, repetition time (TR) = 2300 ms) at the beginning, which was used for anatomical segmentation and registration (see acquisition details in http://adni.loni.usc.edu/methods/documents/)^74^. For rsfMRI acquisition, 140 fMRI volumes were collected with an echo-planar image (EPI) sequence (ADNI-GO and ADNI-2: flip angle = 80^o^, spatial resolution = 3 × 3 × 3 mm^3^, slice thickness = 3.3 mm; see details at: http://adni.loni.usc.edu/methods/documents/) with TR/TE = 3000/30 ms (except for 3 subjects with TR/TE = 2250/30 ms and one subject with TR/TE = 2000/27 ms). The major analyses were also conducted with excluding these four subjects who are in the S1 and S3 subgroups. Aβ-PET data were acquired from the approximately 50 to 70 min post-injection of florbetapir-fluorine-18 (18F-AV-45) (see https://adni.loni.usc.edu/wp-content/uploads/2010/05/ADNI2_PET_Tech_Manual_0142011.pdf).

We followed the previous study^17^ in preprocessing the rsfMRI data with one modification. That is, we excluded the rsfMRI session with large head motion accessed by the session mean (averaged over one fMRI session) frame-wise displacement (FD) larger than 0.5 mm^75–77^. The general procedures for rsfMRI preprocessing include motion correction, skull stripping, spatial smoothing (full width at half maximum (FWHM) = 4 mm), temporal filtering (bandpass filter, 0.01 to 0.1 Hz), and the co-registration of each fMRI volume to corresponding T1-weighted structural MRI and then to the 152-brain Montreal Neurological Institute (MNI-152) space, but not include the step of motion parameter regression to avoid attenuating the gBOLD signal (see the detailed explanation and preprocessing steps at the previous studies^17,53^). The first 5 and last 5 rsfMRI volumes were discarded to ensure a steady magnetization and to avoid the edge effect from the temporal filtering. The major analyses were also repeated with discarding the first 10 and last 10 volumes instead as a sensitivity analysis (Fig. S9). The parcel-based rsfMRI was derived by averaging the preprocessed rsfMRI signal within each of the 68 cortical parcels (DKT-68 parcellation^78^). Both the parcel-based and the voxel-based rsfMRI were kept for the subsequent analyses. The imaging preprocessing was performed by combining FSL (the FMRIB Software Library. ver 5.0.9) and AFNI (Analysis of Functional NeuroImages; ver 16.3.05) software packages for neuroimaging processing. All other data analyses were performed using MATLAB R2019b (MathWorks, Natick, MA, USA).

We directly used the PET-Aβ SUVR data summarized in “UC Berkeley—AV45 Analysis [ADNI1, GO, 2, 3] (version: 2019-07-28)”^71,72^. The major preprocessing procedures for this PET-Aβ data included the florbetapir images averaging, spatial smoothing, and registration to the space of structural MRI to extract the mean Aβ at the gray matter and each cortical parcel (DKT-68 parcellation^78^). The composite region was used as the reference, including the eroded cortical white matter, brainstem/pons, and whole cerebellum^79^. The global (or the regional) Aβ SUVR were respectively calculated as the ratio of the mean florbetapir uptake at the gray matter (or that at each parcel) and the composite reference region. Moreover, a 2-year longitudinal change of the global (or the regional) brain Aβ SUVR was then calculated by subtracting the cortical (or regional) Aβ SUVR at baseline from that at the 2-year follow-up.

### The extraction of CSF inflow signal and the rsfMRI at global and regional brain

We derived the gBOLD signals by averaging the rsfMRI signal across all voxels in the gray-matter region (see a representative example in Figure S1A, left; corresponding to the signal in Figure S1A, middle, green), similar to the previous study^17^. To be specific, we defined gray matter masks based on the Harvard-Oxford cortical and subcortical structural atlases (https://neurovault.org/collections/262/). We used the preprocessed fMRI in the MNI-152 space that went through the above preprocessing procedures (without nuisance regression; the CSF was especially not regressed out since it is related to the CSF inflow signal of interests), and averaged the rsfMRI signals at gray-matter regions in the MNI-152 space (3 × 3 × 3 mm^3^)^80^. The preprocessed fMRI was also used to extract the signal at each parcel of DKT-68 parcellation^78^. The rsfMRI signal of CSF inflow was extracted following our previous study and using the bottom slices of fMRI acquisition^17^. The fMRI signals were then extracted from and averaged within the individual’s CSF mask (see an exemplary mask in Fig. S1A, right and the corresponding signal in Figure S1A, middle, red), using the preprocessed fMRI in the original individual space (without the spatial registration to the MNI-152 space to avoid spatial blurring from the registration process on such a small region, with the same rationale to a previous study^18^).

### The coupling between the global or local BOLD signal and the CSF signal

We calculated the cross-correlation functions between the gBOLD signal or regional BOLD (at each cortex/parcel) and the CSF inflow signals obtained through the above procedures to quantify their coupling, similar to the previous study^17^. Specifically, we first calculated the cross-correlation functions between gBOLD and CSF signals to access their Pearson’s correlation at different time lags, and then used the correlation at the lag of +3 s, where the negative peak of the mean cross-correlation occurred (orange arrow at the Fig. S1B), to quantify the gBOLD-CSF coupling. To be consistent with the gBOLD-CSF coupling, we quantified the regional BOLD-CSF (rBOLD-CSF) coupling using the cross-correlation between the BOLD signal at each cortex/parcel and the CSF signal at the lag of +3 s.

### CSF biomarkers

The baseline data of Aβ42, T-tau, and P-tau in CSF for above 144 subjects were used in the present study. These CSF proteins were measured using the multiplex xMAP Luminex platform (Luminex Corp, Austin, TX, USA) with the INNOBIA AlzBio3 kit (Innogenetics, Ghent, Belgium)^81,82^. We used the median values from multiple batches in the “UPENN CSF Biomarker Master [ADNI1, GO, 2] (version: 2016-07-05)”^73^.

### Stages classifications

Following the previous study^5^, we categorized the entire cohort of subjects into three different groups based on the CSF Aβ42 and cortical Aβ SUVR data at baseline: (1) non-accumulators with normal CSF Aβ42 and normal cortical Aβ (CSF-/PET-; *N* = 50); (2) early Aβ accumulators with abnormal CSF Aβ42 but normal cortical Aβ (CSF+/PET-; *N* = 23, and (3) late Aβ accumulators with abnormal CSF Aβ42 and abnormal cortical Aβ (CSF+/PET + ; *N* = 71). No subjects with normal CSF Aβ42 but abnormal cortical Aβ were found in our cohort. Abnormal CSF Aβ42 was defined with the cut-off of <192 ng/L (as the “CSF+”), and abnormal cortical Aβ subjects were classified based on the cut-off of >0.872 SUVR (as the “PET+”; reference region as the composite area) referring to the previous study^5^.

### Link the gBOLD–CSF coupling to the stage of Aβ accumulation and AD-related protein markers

The gBOLD-CSF coupling strength was evaluated using their cross-correlation at the lag of +3 s. We first compared the gBOLD-CSF coupling (adjusted for age and gender) across the three different Aβ stages described above, including the pairwise comparison (two-sample t-test) and the evaluation of the linear trend changing from the non-accumulators to late Aβ accumulators (S1: CSF-/PET- to S2: CSF+/PET- to S3: CSF+/PET+; ordinal regression).

For each Aβ stage, we correlated the gBOLD-CSF coupling (age and gender adjusted) with the cortical Aβ SUVR at baseline and its changes in 2 years, as well as the natural logarithm of CSF Aβ42, CSF T-tau, and CSF P-tau (Spearman’s rank correlation).

To examine whether and how the CSF protein markers, as well as their associations with the coupling index, are dependent on the PET-Aβ, we correlated them with the cortical Aβ SUVR at baseline and also re-tested the coupling-CSF markers associations after adjusting for this baseline cortical Aβ level.

To understand whether the time lags between gBOLD and CSF signals would vary systematically across different disease conditions (i.e., AD, MCI, and SMC/controls) and affect the gBOLD-CSF coupling strength, we defined the lag for each subject where the gBOLD-CSF cross-correlation function achieves its minimal value and then compared the lags and the corresponding “minimal correlation” across disease conditions.

### Define the lower- and higher-order mask

We defined the higher-order and lower-order brain regions according to Yeo’s 7 networks (derived from 400-Area Parcellation^83^). We regarded DMN and FPN as the higher-order cognitive region whereas the somatomotor (which includes the somatosensory, motor, and auditory regions in Yeo’s 7-network definition^83^) and visual networks as the lower-order sensory-motor region, based on their distinct functional hierarchies^36^. Since the PET-Aβ data were obtained with DKT-68 parcellation^78^, we then assigned the parcels to the higher- and lower-order regions according to the following rule. We classified an individual DKT-68 parcel as a part of the higher-order region if more than 50% vertices of the parcel were overlapped with DMN or FPN, or a part of the lower-order region if it is overlapped more than 50% with the somatomotor and visual networks defined by Yeo’s 7 networks. The generated higher- and lower-order regions from DKT-68 parcels were then binarized and mapped to the brain surface as the higher- and lower-order masks. The parcel labels/names were also listed (Table S1).

### Relate rBOLD-CSF coupling to longitudinal Aβ changes for the early accumulators

For the CSF+/PET- subjects, we computed the 2-year cortical Aβ SUVR changes in all parcels and presented them on the brain surface (also see the 2-year cortical Aβ SUVR changes at the other two stages and the comparison between stages in Figure S4). The rBOLD-CSF coupling at all brain voxels was computed to generate a coupling map for each CSF+/PET- subject. The coupling maps were averaged across the CSF+/PET- subjects to generate the mean coupling map for this group. The map was also projected onto the brain surface using the WorkBench software (version: 1.2.3; https://www.humanconnectome.org/software/workbench-command) with spatial smoothing (Gaussian smoothing, standard deviation of 5 mm). For each CSF+/PET- subject, we also averaged the parcel-based rBOLD-CSF coupling and the 2-year cortical Aβ SUVR changes within the defined higher-order mask. These two measures for the higher-order mask were then correlated with each other across the CSF+/PET- subjects (Spearman’s rank correlation; age and gender were adjusted for the coupling measure). The same procedure was repeated for the lower-order mask, and also for each brain parcel to obtain the region-specific association between 2-year cortical Aβ change and rBOLD-CSF coupling.

### Relate 2-year cortical Aβ changes to gBOLD presence for the early accumulators

For the CSF+/PET- subjects, we also quantified the contribution of the gBOLD signal to the rBOLD signal at each voxel/parcel through their correlation (Spearman’s correlation). We regarded this correlation as a quantification of the gBOLD presence at individual voxels/parcels following a previous study^29^. The gBOLD presence results were averaged across the CSF+/PET- subjects and also projected to the brain surface, similar to the rBOLD-CSF coupling map. The mean gBOLD presence was spatially correlated with the (surface-smoothed) mean rBOLD-CSF coupling across cortices. To quantify the extent to which the Aβ is preferentially accumulated in the higher-order region as compared to the lower-order region, we took the lower-order region as an internal reference and computed the cross-hierarchical contrast for the 2-year changes of cortical Aβ SUVR for each CSF+/PET- subject. Specifically, we averaged the 2-year Aβ accumulation values within the higher-order mask and the lower-order mask, respectively, and then subtracted the latter from the former to generate the cross-hierarchical contrast of 2-year Aβ accumulation for each CSF+/PET- subject. We then correlated (Spearman’s correlation) the cross-hierarchical contrast of 2-year Aβ change with the gBOLD presence (adjusted for age and gender) at each DKT-68 parcel across all the CSF+/PET- subjects. The correlation coefficient at each cortical parcel was then mapped onto the brain surface and spatially smoothed with a Gaussian kernel (standard deviation as 5 mm). We computed the cross-hierarchy contrast metric for the gBOLD presence in a similar way and then correlated it with the cross-hierarchy contrast of 2-year cortical Aβ change across the CSF+/PET- subjects. We also repeated the analyses using DKT-68 parcels segmented for individual subjects in their native imaging space, which had little effect on the coupling quantifications and the major results.

### Correlate the CSF Aβ42 with the connectivity at lower- or higher-order regions

We first computed the functional connectivity (FC) within the higher-order region using the BOLD signals of the higher-order DKT-68 parcels (averaged BOLD within each parcel, Table S1). Two versions of the BOLD signal were used, one with and one without the gBOLD signal being regressed out from the preprocessed rsfMRI signals. The other nuisance variables, including the CSF signal, white matter signal, and head motion parameters, were also regressed out before computing FC. For each CSF+/PET- subject, the Pearson’s correlations of the BOLD signals were computed to measure the FC of all possible pairs of higher-order DKT-68 parcels, which were then averaged to generate a single FC value to represent the FC of the higher-order region. The natural logarithm of CSF Aβ42 was then correlated (Spearman’s correlation), across the early accumulators, with the FC (adjusted for age and gender) within higher-order parcels computed with or without gBOLD signal. The FC within the lower-order region and between higher- and lower-order parcels were also computed and correlated with the CSF Aβ42 in a similar way.

### Quantify the gBOLD propagating waves

The rsfMRI BOLD signals were projected onto the direction of the principal gradient (PG) of brain functional connectivity^36^ to obtain time-position graphs using a method detailed in a previous study^30^. This PG map was generated in a previous study by applying a low-dimensional embedding method (i.e., diffusion mapping) to the mean connectivity matrix from 820 human subjects^36^. First, we sorted cortical voxels according to their PG values and divided them into 70 position bins of equal size^30^. The rsfMRI BOLD signals were temporally interpolated with a scalar value of 5 (increased the sampling rate by 5 folds to a temporal resolution of 0.6 s), averaged within each bin, and displayed as a time-position graph with one dimension representing time and the other denoting the 70 bins along the PG direction. To identify the gBOLD propagating waves, we cut the time-position graph into multiple segments according to troughs of the gBOLD signal^30^. For each time segment, we identified the local BOLD peak of each bin and computed the timing relative to the global mean peak (i.e., gBOLD peak). The relative timing of the local BOLD peak was then correlated with the position of its corresponding bin along the PG direction. A strong positive time-position (Pearson’s) correlation suggested the propagation of the local BOLD peaks from the lower-order sensory regions to the higher-order DMN regions along the PG direction (“bottom-up”), whereas a strong negative time-position correlation indicated the propagation in the opposite direction (“top-down”). The time–position correlation was only calculated for time segments whose local peaks were identified in more than 50 position bins, which account for the majority (71.4%) of the time segments. We then used a correlation threshold of 0.3 (corresponding to the *p*-value of 0.01) to identify the SM-to-DMN (bottom-up, time-position correlation >0.3) and DMN-to-SM (top-down, time-position correlation <−0.3) for each CSF+/PET- subject.

Among the 23 early accumulators, we selected the top and bottom one-third (8 in each) with the highest and lowest CSF Aβ42 and compared their propagations. We aligned and averaged the identified propagations (bottom-up or top-down) with a time window of 12 s centering on their corresponding gBOLD peak (set as time zero). This generated the mean pattern of the propagations. We selected three representative time points at −3 s, 0 s, and +3.6 s (relative to the gBOLD peak) and projected the mean fMRI maps at these time points back to the brain surface to intuitively show the spatial patterns at different phases. The two-sample *t*-test was then employed to compare the time-position graphs and the spatial BOLD maps from the two sub-groups with different levels of CSF Aβ42. The above procedures were performed separately for the SM-to-DMN and DMN-to-SM propagations.

### Statistical analysis

Group comparisons for continuous measures were performed using the two-sample t-test, including age, gBOLD-CSF coupling, cortical Aβ SUVR changes, and each element of time-position graphs between different sub-groups with higher- or lower-level of CSF Aβ42. We used the Fisher exact test^84^ for the comparison of categorical measures (i.e., gender) between stages of Aβ pathology progression (non-accumulators, early accumulators, and late accumulators). The linear trend of gBOLD-CSF coupling changes across the three stages was quantified with the ordinal regression test. Spearman correlation was employed to evaluate inter-subject associations between different variables, such as the link between the rBOLD-CSF coupling and 2-year SUVR change at higher-order regions, due to their non-Gaussian distribution^85^. A slope comparison test (CompareSlopes function in the FMAToolbox; https://fmatoolbox.sourceforge.net/Contents/index.html) was applied to examine whether the regression slopes of CSF Aβ42 and connectivity changed significantly before and after gBOLD removal. In the study, a p-value less than 0.05 was considered statistically significant, and the false discovery rate (FDR) was used for correcting multiple comparisons, e.g., the q-values in correlation analyses in Figs. 1C–E, 2C, and 4A–C.

To test the effect of head motion on our major results, we first examined whether mean FD^86^ changes systematically among the three different Aβ stages and is associated with systematic changes in the gBOLD-CSF coupling. We further repeated the major analyses by regressing out the mean FD from the fMRI-based measures.

To examine whether the major results are held for cognitively unimpaired (control and SMC) subjects, we also repeated the major analyses with excluding the AD and MCI subjects.

To test the effect of physiological function (especially the pulse pressure) on our major results, we also repeated our major analyses by regressing out the mean pulse pressure, i.e., systolic blood pressure minus diastolic blood pressure, from the fMRI-based measures. All statistical tests conducted in this study were two-sided.

### Reporting summary

Further information on research design is available in the Nature Portfolio Reporting Summary linked to this article.

### Supplementary information

Supplementary Information

Reporting Summary

### Source data

Source data

## Data Availability

The multimodal data, including subject characteristics, Aβ42, T-tau, and P-tau in CSF, rsfMRI, and amyloid-PET SUVR, are all publicly available at the ADNI website upon the approval of the data use application (http://adni.loni.usc.edu/). The relevant data from each of the main and supplementary figures have been summarized into a source data file and published along with this paper. The ADNI was launched in 2003 as a public-private partnership, led by Principal Investigator Michael W. Weiner, MD. The primary goal of ADNI has been to test whether serial magnetic resonance imaging (MRI), positron emission tomography (PET), other biological markers, and clinical and neuropsychological assessment can be combined to measure the progression of mild cognitive impairment (MCI) and early Alzheimer’s disease (AD). For up-to-date information, see www.adni-info.org. The files of “UC Berkeley—AV45 Analysis [ADNI1, GO, 2, 3] (version: 2020-05-12)” and “APOE—Results [ADNI1, GO, 2, 3] (version: 2013-05-14)” summarized by ADNI were used to provide the Aβ-PET SUVR and APOE genotype data for the present study. CSF Aβ42, CSF T-tau, and CSF P-tau data for our cohort were obtained from the “UPENN CSF Biomarker Master [ADNI1, GO, 2] (version: 2016-07-05)”. The principal gradient template was obtained from “hcp.embed.all.179.lh.dscalar.nii” and “hcp.embed.all.179.rh.dscalar.nii” at https://github.com/NeuroanatomyAndConnectivity/gradient_analysis/tree/master/gradient_data/templates. The Harvard-Oxford cortical and subcortical structural atlas (https://neurovault.org/collections/262/) was used to derive gray matter mask. DKT-68 atlas was obtained from https://surfer.nmr.mgh.harvard.edu/fswiki/CorticalParcellation. Source data are provided with this paper.

## Code availability

Codes used to derive all results are provided at https://github.com/feng5132308003/Resting-state-global-brain-activity-affects-early--amyloid-accumulation-in-default-mode-network_NEW.git. Further detailed code used in the present study are available from the corresponding author upon request.
