# Supplementary material for: Resting-state global brain activity affects early β-amyloid accumulation in default mode network
Source: Nat Commun. 2023 Nov 27;14:7788. doi: 10.1038/s41467-023-43627-y (PMC10682457; doi:10.1038/s41467-023-43627-y)
Supplement: Supplementary file 1 — Supplementary Information [file 41467_2023_43627_MOESM1_ESM.pdf]

# Supplementary Materials for

## Resting-state global brain activity affects early β-amyloid accumulation in default mode network

Feng Han, Xufu Liu, Richard B. Mailman, Xuemei Huang, Xiao Liu*

*Correspondence to: xxl213@psu.edu

† A list of authors and their affiliations appears at the end of the paper

**This PDF file includes:**

Tables S1

Figs. S1 to S14

## SUPPLEMENTARY TABLE:

**Table S1. Higher-order and lower-order parcels**

| **Higher-order** | **Lower-order** |
|---|---|
| Bankssts, left | Cuneus, left |
| Caudal middle frontal gyrus, left | Fusiform gyrus, left |
| Inferior parietal lobe, left | Lateral occipital cortex, left |
| Isthmus of cingulate gyrus, left | Lingual, left |
| Middle temporal gyrus, left | Parahippocampal gyrus, left |
| Pars opercularis, left | Paracentral lobule, left |
| Pars orbitalis, left | Pericalcarine cortex, left |
| Pars triangularis, left | Postcentral gyrus, left |
| Posterior cingulate cortex, left | Precentral gyrus, left |
| Precuneus, left | Transverse temporal gyrus, left |
| Rostral anterior cingulate cortex, left | Cuneus, right |
| Rostral middle frontal gyrus, left | Fusiform gyrus, right |
| Superior frontal gyrus, left | Lateral occipital cortex, right |
| Bankssts, right | Lingual, right |
| Caudal middle frontal gyrus, right | Parahippocampal gyrus, right |
| Inferior parietal lobe, right | Paracentral lobule, right |
| Isthmus of cingulate gyrus, right | Pericalcarine cortex, right |
| Middle temporal gyrus, right | Postcentral gyrus, right |
| Pars orbitalis, right | Precentral gyrus, right |
| Pars triangularis, right | Transverse temporal gyrus, right |
| Posterior cingulate cortex, right | |
| Precuneus, right | |
| Rostral anterior cingulate cortex, right | |
| Rostral middle frontal gyrus, right | |
| Superior frontal gyrus, right | |

See detailed DKT-68 parcel[1] information at

https://surfer.nmr.mgh.harvard.edu/fswiki/FsTutorial/AnatomicalROI/FreeSurferColorLUT.

# SUPPLEMENTARY FIGURES:

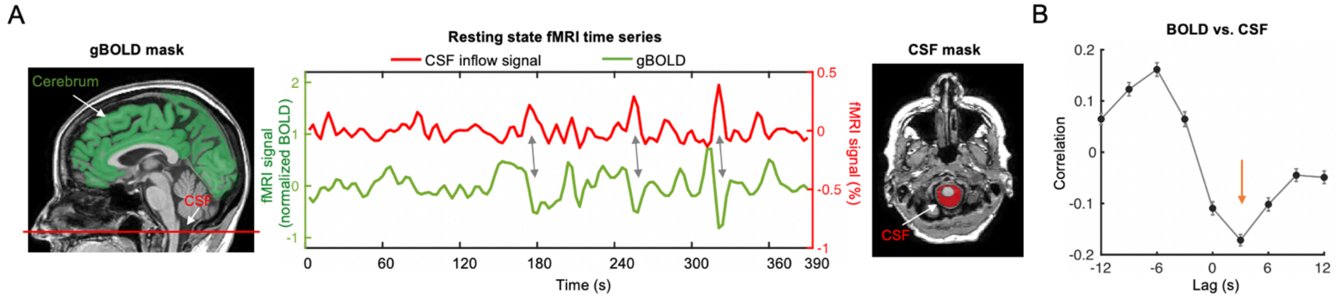


**Figure S1 Global BOLD (gBOLD) signal is coupled with cerebrospinal fluid (CSF) changes.** (A) **Left**: The gBOLD signal was averaged across the signal at all cerebral voxels with excluding the ventricle ones (the green mask on an exemplary structural MRI); **Right**: the CSF inflow signal was extracted from the CSF regions at the bottom slice of the fMRI acquisition (red mask in the T1-weighted MRI; corresponding to the bottom slice of fMRI acquisition, red line in the left panel). **Middle**: The gBOLD signal and the CSF inflow signal from a representative subject showed corresponding changes (gray arrows indicating the synchronization). (**B**) The cross-correlation function between the gBOLD signal and the CSF inflow signal averaged across 144 subjects. Error bar represents one standard error of the mean (SEM). These cross-correlation functions pattern was similar to those reported in the previous studies[2,3]. The cross-correlation at the +3-second lag (orange arrow; subject-mean as -0.17), which also showed the strongest negative coupling in the previous study[2], was used for quantifying the gBOLD–CSF coupling in the present study. The main purpose of this figure is to illustrate the process of deriving the gBOLD-CSF coupling, and it showed similar results as the previous study[2] on an overlapped cohort of subjects. The 83 of 144 subjects in the current study was among the 118 subjects studied there. Source data are provided as a Source Data file.

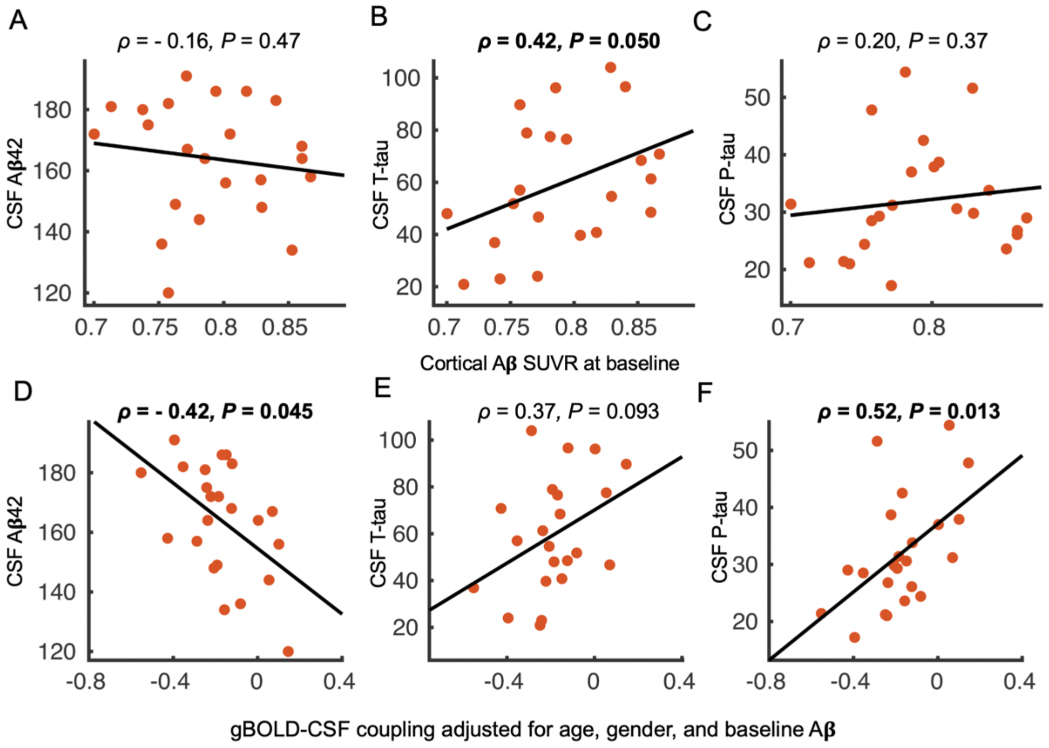


**Figure S2**. (**A-C**) The association between cortical amyloid-beta (Aβ) level and the three cerebrospinal fluid (CSF) markers across S2: CSF+/PET- subjects ($N$ = 23). (**D-F**) The coupling-CSF markers association remained strong after regressing the baseline Aβ from the coupling metrics. Two-sided Spearman's correlation analysis was used in each panel. Source data are provided as a Source Data file.

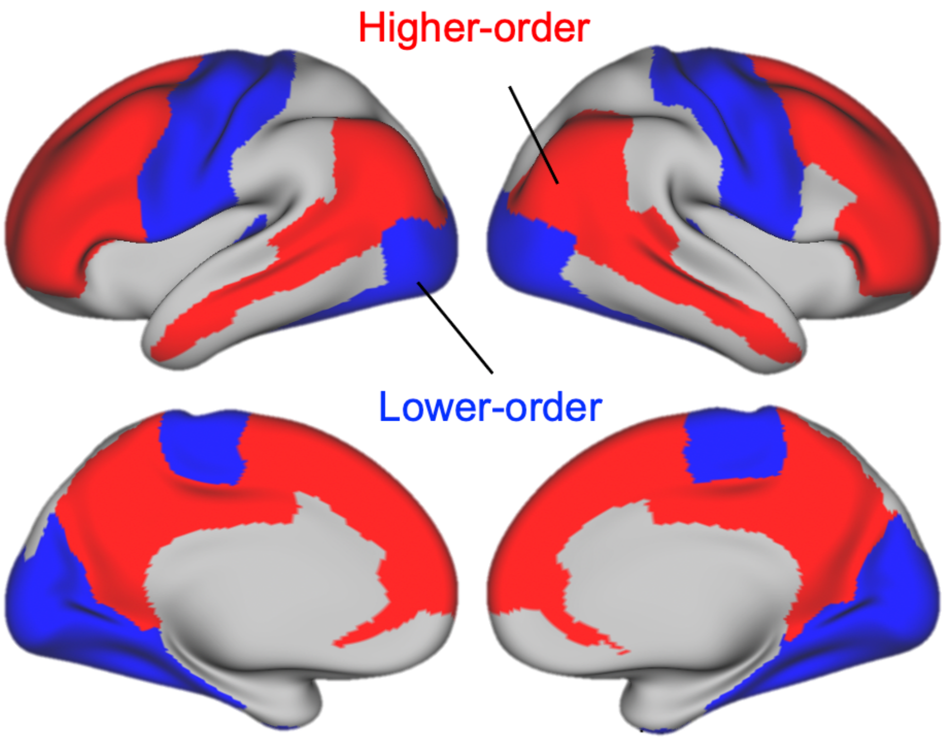


**Figure S3 Higher- and lower-order masks.** We derived the higher-order mask consisting of the DKT-68 parcels[1] belonging to default mode network (DMN) and frontoparietal network (FPN), and the lower-order mask including those the somatosensory and visual parcels. These networks were defined by Yeo's 7 network parcellation[4] (refer to **Methods** for details).

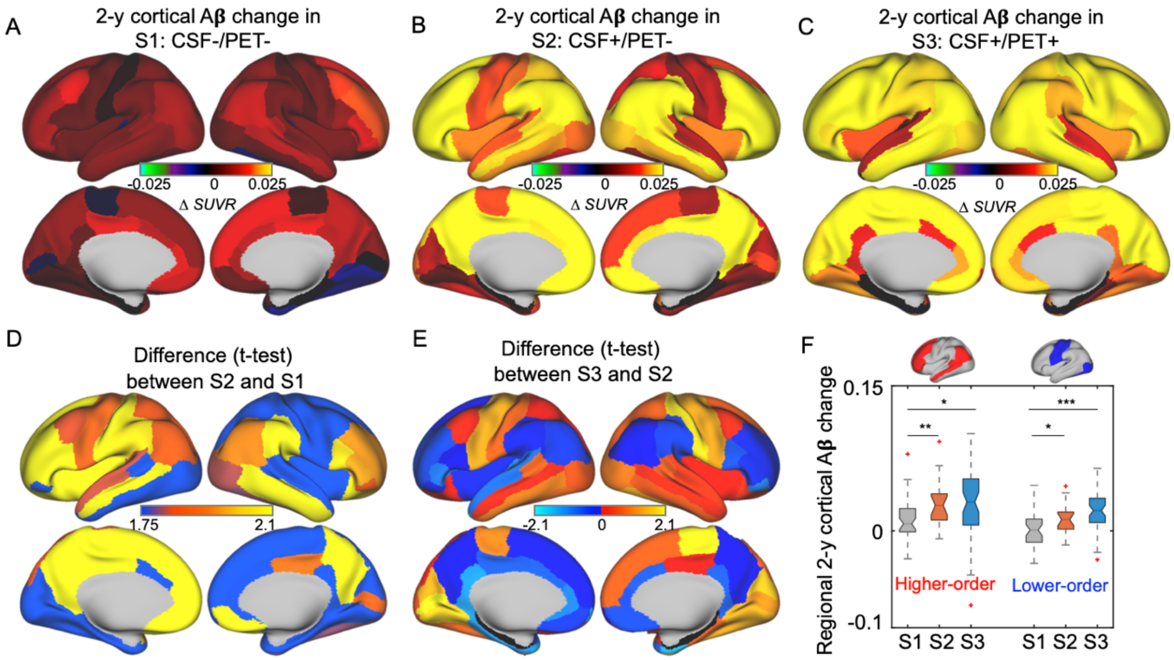


**Figure S4 Surface maps of two-year cortical amyloid-beta (Aβ) change at each stage of Aβ pathology progression.** (**A-C**) Group-mean cortical Aβ change in 2 years at each DKT-68 parcel (adjusted for age and gender) for S1: CSF-/PET- (**A**; $N = 42$), S2: CSF+/PET- (**B**; $N = 19$), and S3: CSF+/PET+ (**C**; $N = 51$). (**D-E**) We applied the two-sample t-test (two-sided) between the stage S2: CSF+/PET- and S1: CSF-/PET- (**D**), as well as between S3: CSF+/PET+ and S2: CSF+/PET- (**E**) ($t = 2.1$ corresponding to $P = 0.05$). These between-stage differences were consistent with the previous study[5] that the cortical Aβ accumulated more rapid at the higher-order regions from S1: CSF-/PET- to S2: CSF+/PET- and then at the lower-order networks from S2: CSF+/PET- to S3: CSF+/PET+. (**F**) We also extracted the cortical Aβ accumulated at higher- or lower-order masks (see **Figure S3**) to quantitatively compare the cortical Aβ changes at the two masks (adjusted for age and gender; two-sample t-test, two-sided). The notch box plot showed the cortical Aβ at higher-order regions accumulated more from the first stage to the second stage ($P < 0.01$), while the Aβ at lower-order ones increased more steadily across stages (The sample size for S1, S2, and S3 are $N = 42$, $N = 19$, and $N = 51$, respectively). The bottom and top edges and the central line of the boxes represent the first and third quartiles and the median respectively, and the whiskers represent the minimum and maximum. The "notches" on the boxes delineate the 95% conference interval for the median. Asterisks represent significant level (*: $0.01 < P < 0.05$; **: $0.001 < P < 0.01$; and ***: $P < 0.001$). Source data are provided as a Source Data file.

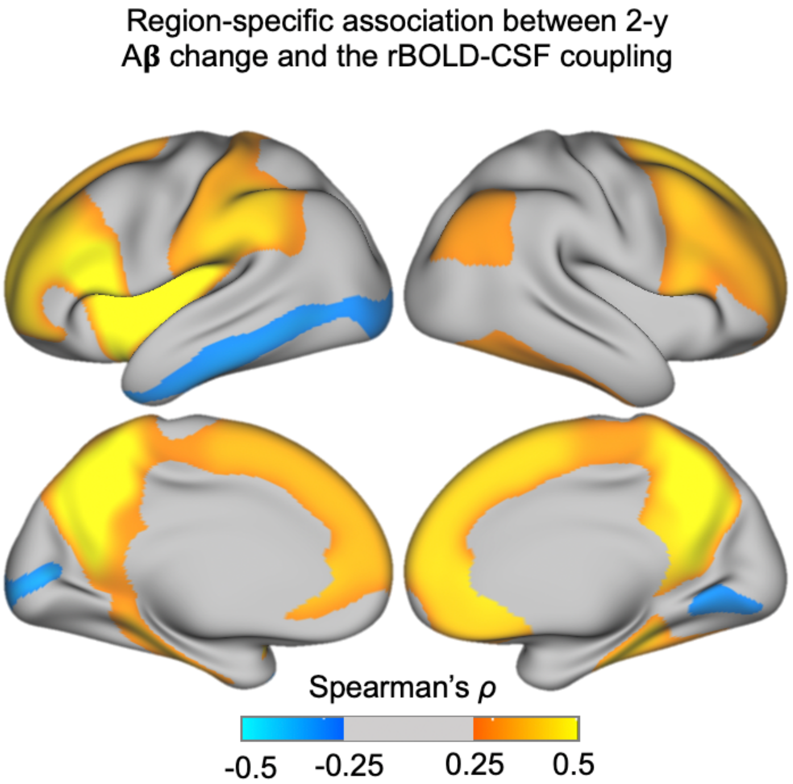


**Figure S5 Region-specific association between two-year amyloid-beta (Aβ) change and the rBOLD-CSF (regional BOLD-cerebrospinal fluid) coupling.** For each of DKT-68 parcels[1], we correlated the two-year Aβ change with the region-specific BOLD-CSF coupling (adjusted for age and gender; two-sided) across and found a strong positive correlation at the higher-order regions ($N = 19$; blue: Spearman's $\rho \leqslant -0.25$; yellow: $\rho \geqslant 0.25$, gray: $-0.25 < \rho < 0.25$).

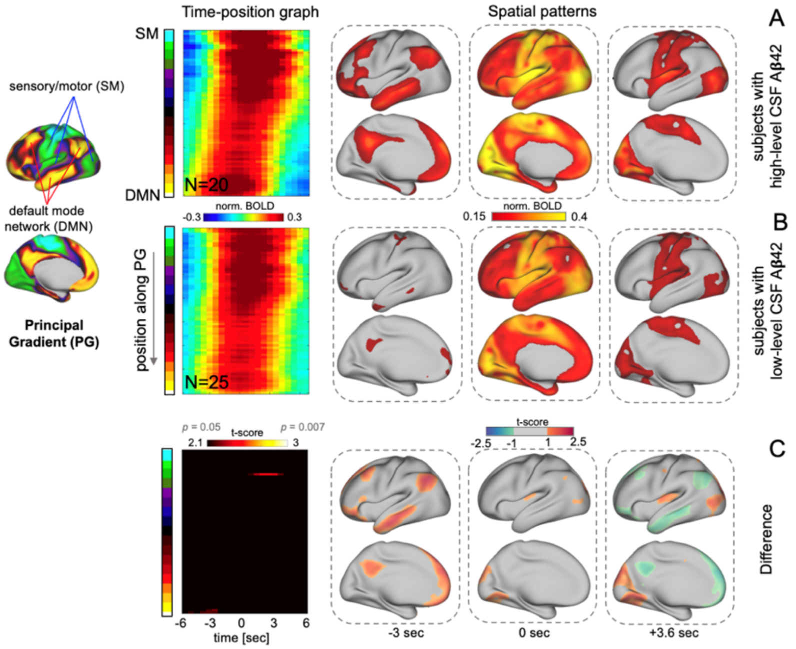


**Figure S6 Early accumulators with distinct level of cerebrospinal fluid (CSF) amyloid-beta 42 (Aβ42) have different default mode network to sensory-motor (DMN-to-SM) propagation pattern of the global brain activation.** (**A-B**) Similar to **Figure 5**, we obtained the mean time-position graphs of activation propagation for the two extreme groups with highest (8 subjects; mean of 20 segments) or lowest (8 subjects; mean of 25 segments) CSF Aβ42. The tilted bands (top-left panel) showed the segment-mean **DMN-to-SM** pattern that propagates from the higher-order DMN regions (align the principal gradient (PG)) to the lower-order somatosensory network. The detailed spatial patterns of cortical co-activation at three representative temporal phases are shown in the right panels (within the dashed rounded rectangles). Both the time-position graphs and spatial maps from the two sub-groups showed a much weaker activation at the higher-order DMN in low-level CSF Aβ42 subjects at the early propagation phase. (**C**) A two-sample t-test (two-sided) was used to test the difference between the **DMN-to-SM** propagation segments from the highest and lowest CSF Aβ42 subjects. The results showed the highest CSF Aβ42 subjects had stronger higher- (DMN) and lower-order activation at around -3s and +3.6s, respectively, but these high-level CSF Aβ42 subjects appeared to be with weaker DMN activation at +3.6s compared with low-level CSF Aβ42 ones (bottom-right panel). The hot color in the bottom-left panel showed that the activation in the high-level CSF Aβ42 subjects was significantly stronger ($P < 0.05$, i.e., $t > 2.1$) than that in the low-level CSF Aβ42 ones. Source data are provided as a Source Data file.

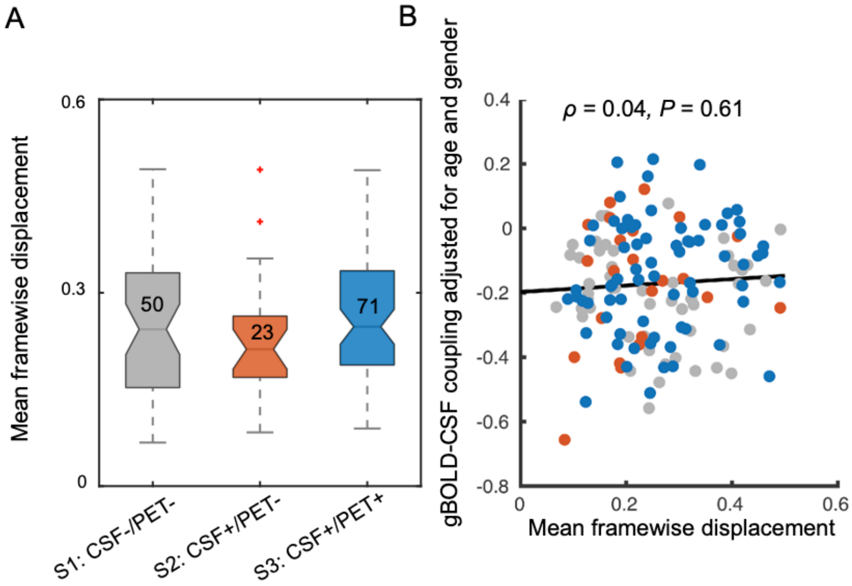


**Figure S7 Head-motion measures change with amyloid (Aβ) stages and gBOLD-CSF (global BOLD–cerebrospinal fluid) coupling.** (**A**) The session-based head motion quantification, mean FD, showed no significant changes across different Aβ stages (two-sided two-sample t-test). The bottom and top edges and the central line of the boxes represent the first and third quartiles and the median respectively, whereas the whiskers represent the minimum and maximum (excluding outliers that are shown as red plus symbols). The "notches" on the boxes delineate the 95% conference interval for the median. The number of subjects in each subgroup is shown on the boxes. The sample size of each subgroup is shown on the boxes. (**B**) The mean frame-wise displacement (FD) was not associated with gBOLD-CSF coupling measures ($N$ = 144; two-sided Spearman's correlation analysis). Source data are provided as a Source Data file.

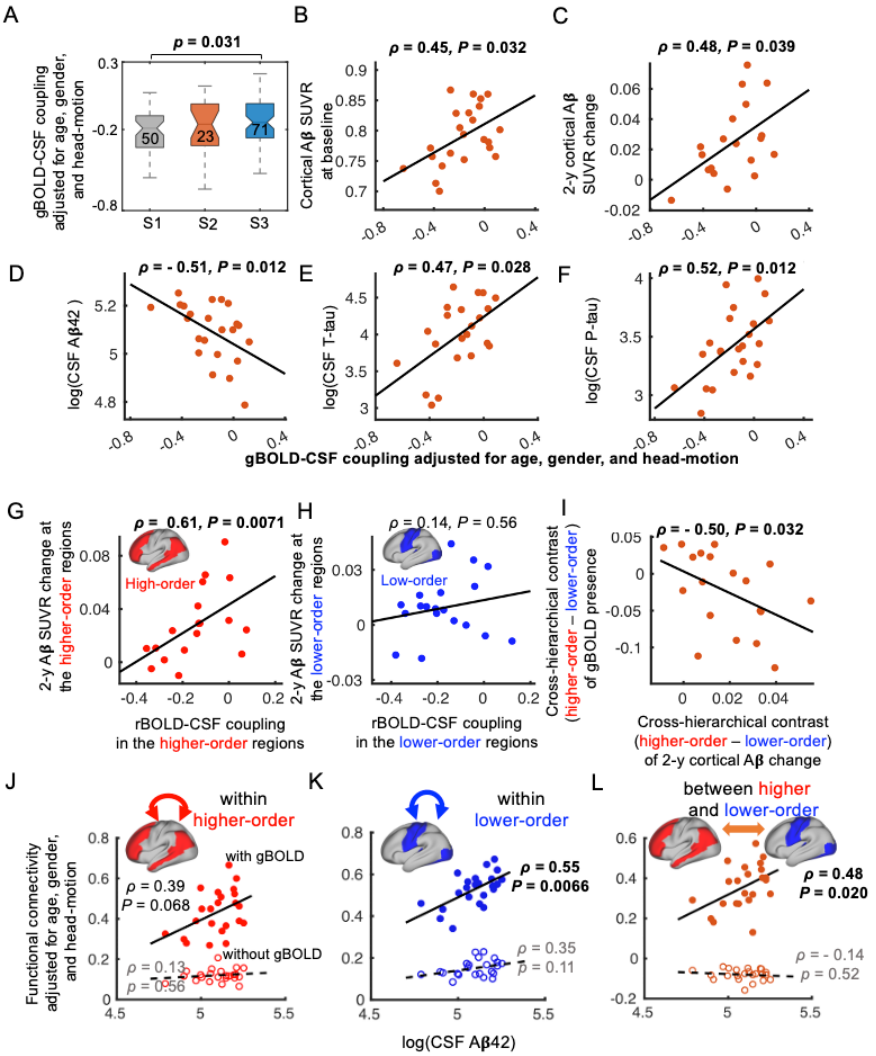


**Figure S8 The associations between fMRI measures and various Alzheimer's disease (AD) protein markers with controlling head motion.** (**A-F**) We re-tested the association between the coupling index and amyloid-beta (Aβ) stages or various AD protein markers shown in the **Figure 1** after regressing out the head motion quantified by the mean frame-wise displacement (FD) of subjects. All associations remained similar and significant. The bottom and top edges and the central line of the notch boxes represent the first and third quartiles and the median respectively, amd the whiskers represent the minimum and maximum. The "notches" on the boxes delineate the 95% conference interval for the median. The sample size of each subgroup is shown on the notch boxes. Two-sided t-test and Spearman's correlation analyses were used. (**G-L**) We also repeated the major analyses presented in **Figure 2C, 3D, and 4** (two-sided) with controlling for the mean FD (as well as age and gender), and minimal changes were observed.

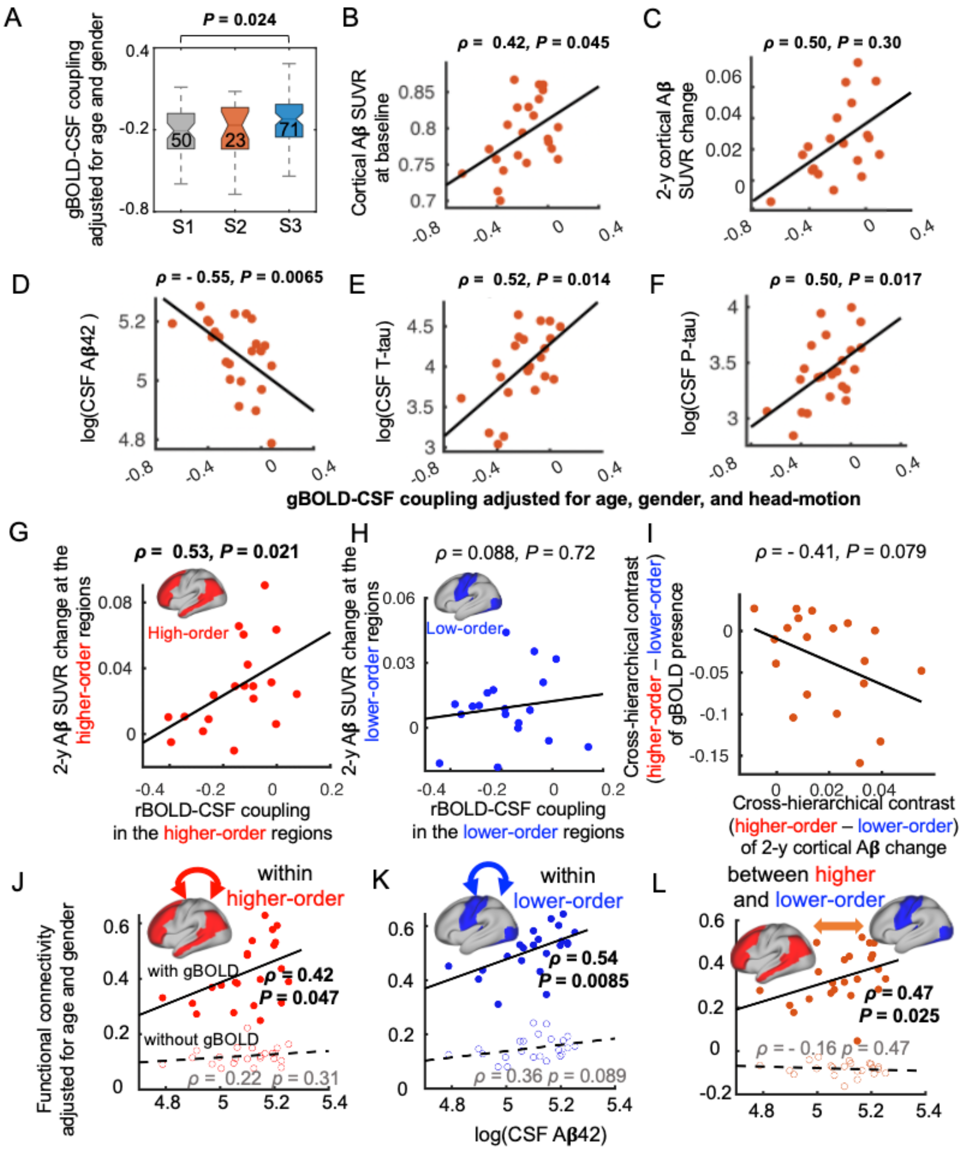


**Figure S9 The associations between fMRI measures and various Alzheimer's disease (AD) protein markers after discarding 10 more fMRI volumes (i.e., first 10 and last 10 volumes excluded).** (**A-F**) We re-tested the association between the coupling index and amyloid-beta (Aβ) stages or various AD protein markers shown in the **Figure 1** after discarding first 10 and last 10 fMRI volumes. All associations remained significant as **Figure 1**. The sample sizes of each subgroup are shown by numbers on the box plots. Two-sided t-test and Spearman's correlation analyses were used. (**G-L**) We also repeated the major analyses presented in **Figure 2C, 3D, and 4** (two-sided) with discarding first 10 and last 10 fMRI volumes, and minimal changes were observed. Source data are provided as a Source Data file.

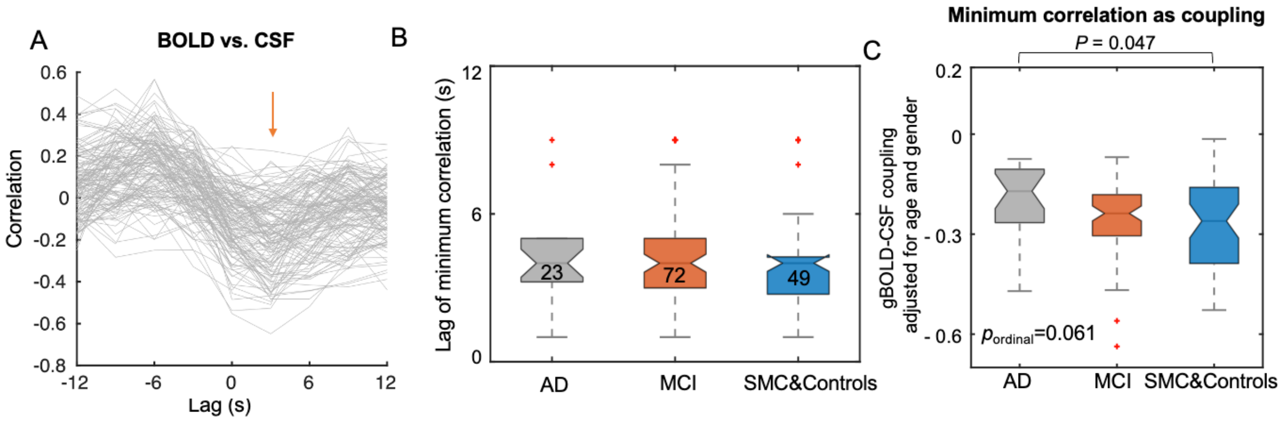


**Figure S10 Subject-specific lags would not either variate among different disease conditions (i.e., Alzheimer's disease (AD), mild cognitive impairment (MCI), and significant memory concern (SMC)/controls) or affect the cross-condition trend of gBOLD-CSF (global BOLD–cerebrospinal fluid) coupling** (**A**) The gBOLD-CSF cross-correlation function of each subject. The orange arrow indicates the +3sec lag, which was used in our main results. (**B**) The time-lags, where the minimums of gBOLD-CSF cross-correlation function occurred, would not significantly change with the different disease severities. The bottom and top edges and the central line of the boxes represent the first and third quartiles and the median respectively, whereas the whiskers represent the minimum and maximum (excluding outliers that are shown as red plus symbols). The "notches" on the boxes delineate the 95% conference interval for the median. The sample size of each subgroup is shown on the notch boxes. (**C**) When the minimum gBOLD-CSF cross-correlations were taken as the new coupling metrics, a little more abrupt decrease was found across the severities than the previous coupling ($p_{ordinal}$ = 0.061; two-sample, two-sided t-test was also applied). Source data are provided as a Source Data file.

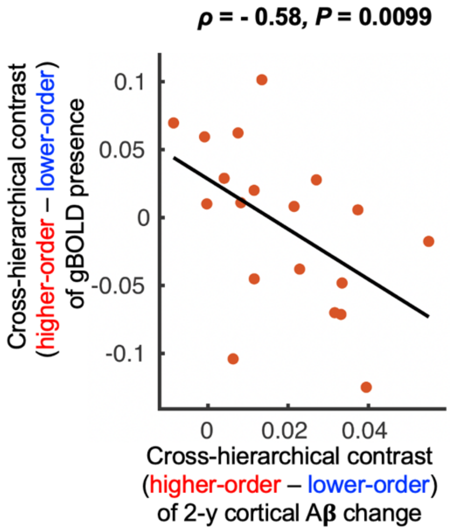


**Figure S11** The replication of major correlation results in **Figure 3D** with extracting the regional BOLD signals in native space for each subject (Two-sided Spearman's correlation). Source data are provided as a Source Data file.

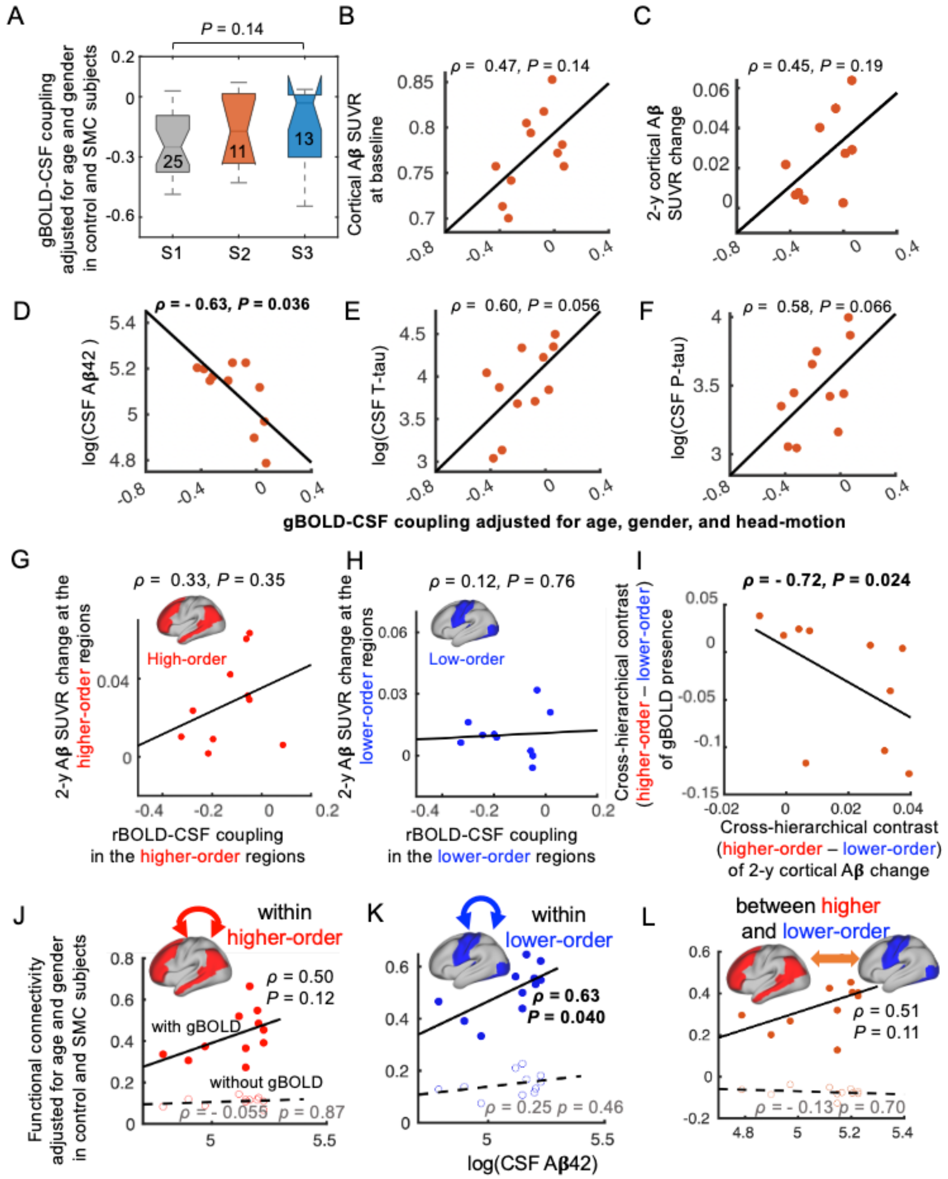


**Figure S12 The associations between fMRI measures and various Alzheimer's disease (AD) protein markers after excluding AD and mild cognitive impairment (MCI) subjects.** (**A-F**) We re-tested the association between the coupling index and amyloid-beta (Aβ) stages or various AD protein markers shown in the **Figure 1** after excluding AD and MCI subjects. All the associations showed very similar pattern as the results from the entire cohort. Some correlations were no more significant, which may result from very limited sample size. The sample sizes of each subgroup are shown by numbers on the box plots. (**G-L**) We also repeated the major analyses presented in **Figure 2C, 3D, and 4** (two-sided) with excluding AD and MCI subjects and found similar results. Source data are provided as a Source Data file.

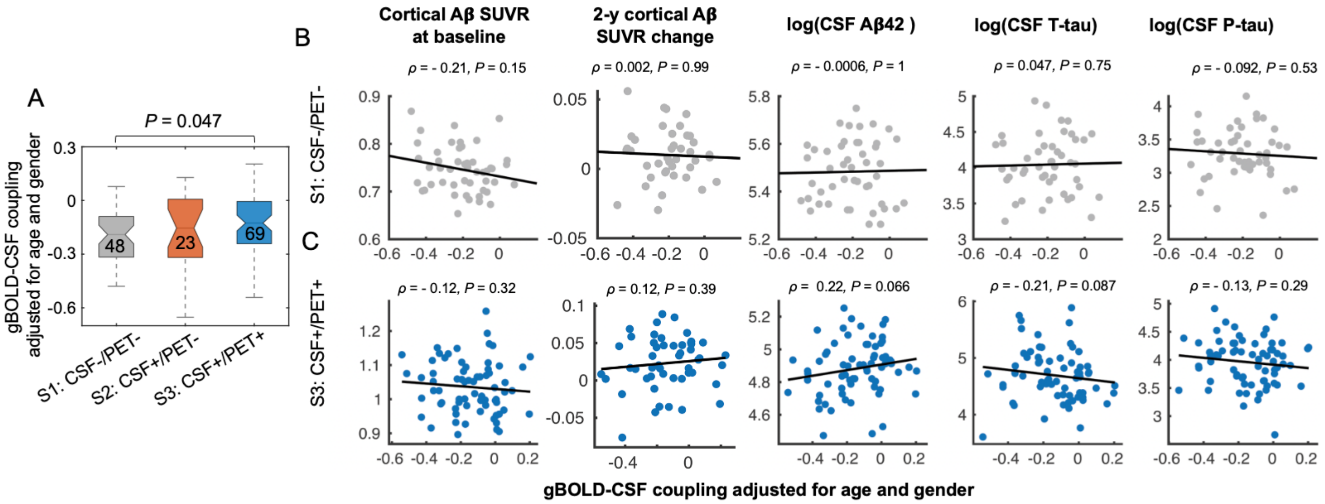


**Figure S13 Excluding the subjects scanned with different repetition time (TRs) and echo time (TEs) make no significant difference on the results in the Figure 1.** There are 4 subjects with shorter TRs, who were in S1: CSF-/PET- or S3: CSF+/PET+. The major results for the Stage 2 subjects would not be affected. We also excluded the 4 subjects, repeated the analyses in **Figure 1**, and found no significant changes of both the coupling-amyloid-beta (Aβ) stages (**A**) and the coupling-marker association results (**B-C**, tests for the slope change before and after exclusion, all $p > 0.96$). The sample sizes of each subgroup are shown by numbers on the box plots. Two-sided t-test and Spearman's correlation analyses were used. Source data are provided as a Source Data file.

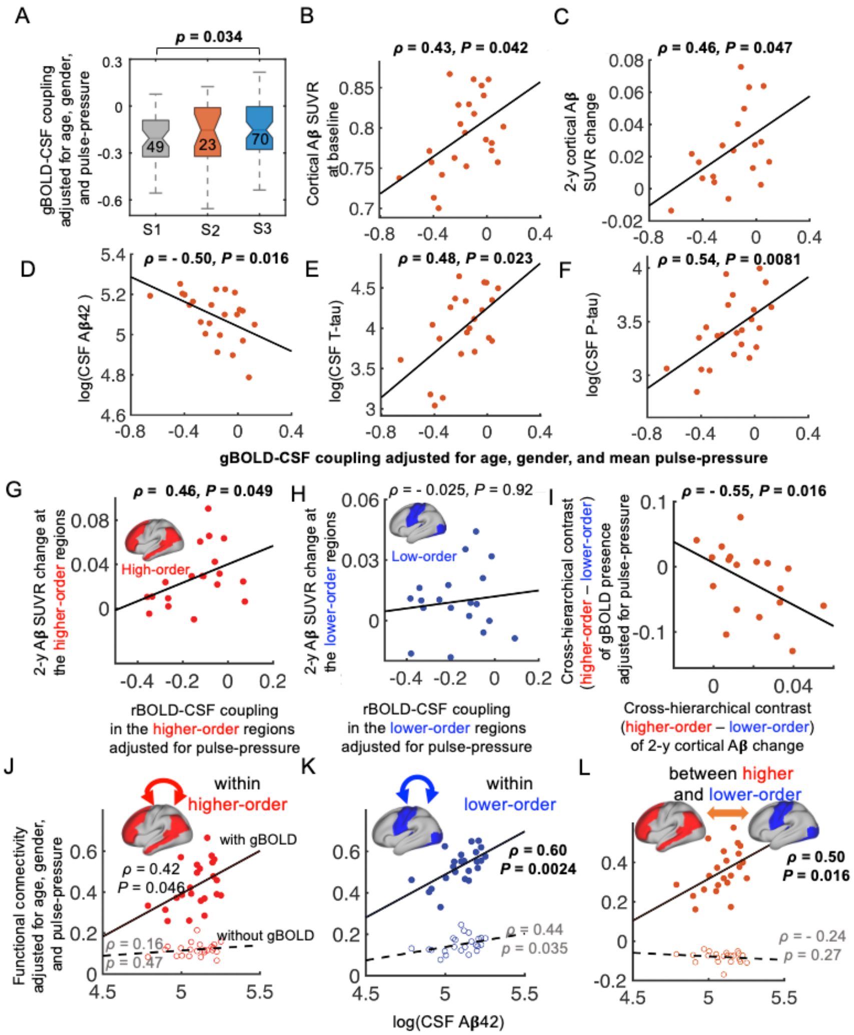


**Figure S14 The associations between fMRI measures and various Alzheimer's disease (AD) protein markers with controlling pulse-pressure.** (**A-F**) We re-tested the association between the coupling index and amyloid-beta (Aβ) stages or various AD protein markers shown in the **Figure 1** after regressing out the pulse-pressure quantified by the systolic blood pressure minus diastolic blood pressure. All associations remained similar and significant. The bottom and top edges and the central line of the boxes represent the first and third quartiles and the median respectively, whereas the whiskers represent the minimum and maximum. The "notches" on the boxes delineate the 95% conference interval for the median. The sample size of each subgroup is shown on the notch boxes. (**G-L**) We also repeated the major analyses presented in **Figure 2C**, **3D**, and **4** with controlling for the pulse-pressure (as well as age and gender), and minimal changes were observed. Two-sided t-test and Spearman's correlation analyses were used. Source data are provided as a Source Data file.

## REFERENCES FOR SUPPLEMENTARY INFORMATION


1. Desikan, R. S. *et al.* An automated labeling system for subdividing the human cerebral cortex on MRI scans into gyral based regions of interest. *Neuroimage* (2006). doi:10.1016/j.neuroimage.2006.01.021
2. Han, F. *et al.* Reduced coupling between cerebrospinal fluid flow and global brain activity is linked to Alzheimer disease–related pathology. *PLoS Biol.* **19**, 1–25 (2021).
3. Fultz, N. E. *et al.* Coupled electrophysiological, hemodynamic, and cerebrospinal fluid oscillations in human sleep. *Science (80-. ).* (2019). doi:10.1126/science.aax5440
4. Schaefer, A. *et al.* Local-Global Parcellation of the Human Cerebral Cortex from Intrinsic Functional Connectivity MRI. *Cereb. Cortex* (2018). doi:10.1093/cercor/bhx179
5. Palmqvist, S. *et al.* Earliest accumulation of β-amyloid occurs within the default-mode network and concurrently affects brain connectivity. *Nat. Commun.* (2017). doi:10.1038/s41467-017-01150-x
