# Supplementary material for: Resting-state global brain activity affects early β-amyloid accumulation in default mode network
Source: Nat Commun. 2023 Nov 27;14:7788. doi: 10.1038/s41467-023-43627-y (PMC10682457; doi:10.1038/s41467-023-43627-y)
Supplement: Supplementary file 2 — Reporting Summary [file 41467_2023_43627_MOESM2_ESM.pdf]

nature portfolio

Corresponding author(s): Xiao Liu

Last updated by author(s): Sep 05, 2023

Reporting Summary

Nature Portfolio wishes to improve the reproducibility of the work that we publish. This form provides structure for consistency and transparency in reporting. For further information on Nature Portfolio policies, see our Editorial Policies and the Editorial Policy Checklist.

Statistics

For all statistical analyses, confirm that the following items are present in the figure legend, table legend, main text, or Methods section.

n/a	Confirmed	
☐	☒	The exact sample size (*n*) for each experimental group/condition, given as a discrete number and unit of measurement
☐	☒	A statement on whether measurements were taken from distinct samples or whether the same sample was measured repeatedly
☐	☒	The statistical test(s) used AND whether they are one- or two-sided<br>*Only common tests should be described solely by name; describe more complex techniques in the Methods section.*
☐	☒	A description of all covariates tested
☐	☒	A description of any assumptions or corrections, such as tests of normality and adjustment for multiple comparisons
☐	☒	A full description of the statistical parameters including central tendency (e.g. means) or other basic estimates (e.g. regression coefficient) AND variation (e.g. standard deviation) or associated estimates of uncertainty (e.g. confidence intervals)
☐	☒	For null hypothesis testing, the test statistic (e.g. *F*, *t*, *r*) with confidence intervals, effect sizes, degrees of freedom and *P* value noted<br>*Give P values as exact values whenever suitable.*
☒	☐	For Bayesian analysis, information on the choice of priors and Markov chain Monte Carlo settings
☒	☐	For hierarchical and complex designs, identification of the appropriate level for tests and full reporting of outcomes
☐	☒	Estimates of effect sizes (e.g. Cohen's *d*, Pearson's *r*), indicating how they were calculated

Our web collection on statistics for biologists contains articles on many of the points above.

Software and code

Policy information about availability of computer code

Data collection	no software was used
Data analysis	MATLAB R2019b. FSL (the FMRIB Software Library) 5.0.9; AFNI (Analysis of Functional NeuroImages) 16.3.05.<br><br>Codes in data analysis are provided at https://github.com/feng5132308003/Resting-state-global-brain-activity-affects-early--amyloid-accumulation-in-default-mode-network_NEW.git

For manuscripts utilizing custom algorithms or software that are central to the research but not yet described in published literature, software must be made available to editors and reviewers. We strongly encourage code deposition in a community repository (e.g. GitHub). See the Nature Portfolio guidelines for submitting code & software for further information.

Data

Policy information about availability of data

All manuscripts must include a data availability statement. This statement should provide the following information, where applicable:

- Accession codes, unique identifiers, or web links for publicly available datasets
- A description of any restrictions on data availability
- For clinical datasets or third party data, please ensure that the statement adheres to our policy

The multimodal data, including subject characteristics, Aβ42, T-tau, and P-tau in CSF, rsfMRI, Amyloid-PET SUVR, in this study have been deposited in the source

data files. The raw de-identified ADNI data are all publicly available at the ADNI website upon the approval of the data use application (http://adni.loni.usc.edu/). Source data are provided with this paper.
The ADNI was launched in 2003 as a public-private partnership, led by Principal Investigator Michael W. Weiner, MD. The primary goal of ADNI has been to test whether serial magnetic resonance imaging (MRI), positron emission tomography (PET), other biological markers, and clinical and neuropsychological assessment can be combined to measure the progression of mild cognitive impairment (MCI) and early Alzheimer's disease (AD). For up-to-date information, see www.adni-info.org.
The files of "UC Berkeley—AV45 Analysis [ADNI1, GO, 2, 3] (version: 2020-05-12)" and "APOE—Results [ADNI1, GO, 2, 3] (version: 2013-05-14)" summarized by ADNI were used to provide the Aβ-PET SUVR and APOE genotype data for the present study. CSF Aβ42, CSF T-tau, and CSF P-tau data for our cohort were obtained from the "UPENN CSF Biomarker Master [ADNI1, GO, 2] (version: 2016-07-05)".
The principal gradient template was obtained from "hcp.embed.all.179.lh.dscalar.nii" and "hcp.embed.all.179.rh.dscalar.nii" at https://github.com/NeuroanatomyAndConnectivity/gradient_analysis/tree/master/gradient_data/templates. The Harvard-Oxford cortical and subcortical structural atlas (https://neurovault.org/collections/262/) was used to derive gray matter mask. DKT-68 atlas was obtained from https://surfer.nmr.mgh.harvard.edu/fswiki/CorticalParcellation.

Research involving human participants, their data, or biological material

Policy information about studies with human participants or human data. See also policy information about sex, gender (identity/presentation), and sexual orientation and race, ethnicity and racism.

Reporting on sex and gender	This study included 71 male and 73 female subjects, and our findings apply to both male and female subjects. The sex and gender information were collected and described by the ADNI project (https://adni.loni.usc.edu/). The sex and gender have been considered in study design. They were also encoded as variables and then regressed out during data analysis.
Reporting on race, ethnicity, or other socially relevant groupings	No applicable socially relevant categorization variables in the manuscript. Increasing diversity recruitment efforts in ADNI may also reflect recent enrollment trends in large databases that will have impacts on AD research in the US.
Population characteristics	This study included 144 participants from the ADNI project (ADNI-GO and ADNI-2) according to the availability of rsfMRI, CSF Aβ42, and 18F-AV45 amyloid PET data. The present cohort consisted of healthy controls (N=28), significant memory concern (SMC; N=21) subjects, mild cognitive impairment (MCI; N=72) subjects, and AD patients (N=23), which were defined by ADNI (http://adni.loni.usc.edu/study-design/). We summarized the participant characteristics, including age, gender, and the number of APOE ε4 allele carrying. The study relevant covariates are described in Table 1.<br><br>To investigate the longitudinal cortical Aβ accumulation, we identified and examined 112 participants, out of the 144, with 2-year follow-up (mean: 24.0, standard deviation: 1.2 months) data of Aβ-PET. No participants in the present study experienced changes in the disease condition over the 2 years.
Recruitment	ADNI patients are recruited in the North American ADNI study from specialized centers that participate in ADNI. Details on ADNI recruitment, inclusion criteria and study design can be found on the ADNI website.<br>No any potential self-selection bias or other biases that we're aware of could potential impact the reported results
Ethics oversight	All participants provided written informed consent. Investigators at each ADNI participating site obtained ethical approval from the individual institutional review board. ADNI data were collected per the principles of the Declaration of Helsinki.<br><br>The use of de-identified data from the ADNI and the sharing of analysis results have been reviewed and approved by the Pennsylvania State University IRB (IRB#: STUDY00014669), and also strictly followed the ADNI data use agreements.

Note that full information on the approval of the study protocol must also be provided in the manuscript.

Field-specific reporting

Please select the one below that is the best fit for your research. If you are not sure, read the appropriate sections before making your selection.

[x] Life sciences [ ] Behavioural & social sciences [ ] Ecological, evolutionary & environmental sciences

For a reference copy of the document with all sections, see nature.com/documents/nr-reporting-summary-flat.pdf

Life sciences study design

All studies must disclose on these points even when the disclosure is negative.

Sample size	ADNI, n = 144. Sample sizes were determined based on maximum available data that met the inclusion criteria. No specific statistical methods or sample size estimations were used to define sample size.
Data exclusions	ADNI has data exclusion criteria in participants screening (see details at https://adni.loni.usc.edu/wp-content/uploads/2008/07/adni2-procedures-manual.pdf). No data were intentionally excluded from our current study.
Replication	All the major analyses were initially done by the first author and the senior author and then independently cross-validated by the second author. We provide access to all code and scripts that we used, and since the ADNI data are publicly available, our findings can be checked.
Randomization	This was an observational study without any intervention, and thus no randomization was performed.

Blinding	This was a study that retrospectively analyzed available observational neuroimaging and clinical data without any intervention. No blinding was performed.

Reporting for specific materials, systems and methods

We require information from authors about some types of materials, experimental systems and methods used in many studies. Here, indicate whether each material, system or method listed is relevant to your study. If you are not sure if a list item applies to your research, read the appropriate section before selecting a response.

Materials & experimental systems

n/a	Involved in the study
☒	☐ Antibodies
☒	☐ Eukaryotic cell lines
☒	☐ Palaeontology and archaeology
☒	☐ Animals and other organisms
☐	☒ Clinical data
☒	☐ Dual use research of concern
☒	☐ Plants

Methods

n/a	Involved in the study
☒	☐ ChIP-seq
☒	☐ Flow cytometry
☐	☒ MRI-based neuroimaging

Clinical data

Policy information about clinical studies

All manuscripts should comply with the ICMJEguidelines for publication of clinical research and a completedCONSORT checklist must be included with all submissions.

Clinical trial registration	NCT00106899 (ADNI) and NCT01231971 (ADNI2).
Study protocol	The ADNI study protocol can be found online at https://adni.loni.usc.edu/methods/documents/.
Data collection	All imaging and clinical data were collected at participating ADNI sites (https://adni.loni.usc.edu/data-samples/).
Outcomes	The study is an observational study that retrospectively analyzed available datasets, so no specific outcomes were defined a priori.

Magnetic resonance imaging

Experimental design

Design type	resting-state fMRI
Design specifications	No specific task was used.
Behavioral performance measures	No behavioral performance was measured during the resting-state fMRI.

Acquisition

Imaging type(s)	functional (and the structural MRI for fMRI processing)
Field strength	3 Tesla
Sequence & imaging parameters	Rs-fMRI: EPI sequence, flip angle = 80o, spatial resolution = 3 × 3 × 3 mm3, slice thickness = 3.3 mm, 48 slices, TR/TE=3,000/30 ms (with the exception of 3 subjects with TR/TE=2,250/30 ms and one subject with TR/TE=2,000/27 ms), 140 volumes.<br><br>T1-weighted MRI: MPRAGE sequence, TE=3.1 ms, TR=2,300 ms, flip angle=9o, voxel size=1.1× 1.1 × 1.2 mm3, FOV=256×240 mm2, 170 slices.
Area of acquisition	Whole brain and cerebrospinal fluid (CSF). The CSF and brain region were determined and defined at the Methods section (See the sub-section of "The extraction of CSF inflow signal and the rsfMRI at global and regional brain").

Diffusion MRI ☐ Used ☒ Not used

Preprocessing

Preprocessing software	FSL 5.0.9; AFNI 16.3.05.
Normalization	T1-weighted images were linearly normalized to the MNI space. After co-registration to structural scans, functional images

	were normalized to the MNI space by applying parameters from the T1-weighted images.
Normalization template	MNI template: ICBM152
Noise and artifact removal	We excluded the rsfMRI session with large head-motion accessed by the session-mean frame-wise displacement; motion correction, skull stripping, spatial smoothing (full width at half maximum (FWHM) = 4mm), temporal filtering (bandpass filter, 0.01 to 0.1 Hz) were applied; the first 5 and last 5 rsfMRI volumes were discarded to ensure a steady magnetization and to avoid the edge effect from the temporal filtering.<br><br>To test the effect of head-motion on our major results, we repeated the major analyses with regressing out the session-mean frame-wise displacement from the fMRI-based measures.<br><br>The nuisance variables, including the CSF signal, white matter signal, and head motion parameters, were regressed out before computing functional connectivity.
Volume censoring	The first 5 and last 5 rsfMRI volumes were discarded to ensure a steady magnetization and to avoid the edge effect from the temporal filtering.

Statistical modeling & inference

Model type and settings	fMRI measures or PET-amyloid data was used as dependent variables in the statistical analysis.
Effect(s) tested	Group comparisons for continuous measures were performed using the two-sample t-test; Fisher exact test was used for the comparison of categorical measures; Spearman's correlation (coefficient and p-value) was employed to evaluate inter-subject associations between different variables. (See "Statistical analysis" in Methods section for details)

Specify type of analysis: ☐ Whole brain ☐ ROI-based ☒ Both

Anatomical location(s): DKT-68 parcellation (Freesurfer ROIs; see Methods for details)

Statistic type for inference<br>(See Eklund et al. 2016)	No voxel-wise or cluster-wise statistical analysis was used in our main results (Fig.1, Fig.2C, Fig.3D, Fig.4, and Fig.5).
Correction	No analyses involved multiple comparisons.

Models & analysis

n/a	Involved in the study
☐	☒ Functional and/or effective connectivity
☒	☐ Graph analysis
☒	☐ Multivariate modeling or predictive analysis

Functional and/or effective connectivity	Pearson's correlation was used to derive the functional connectivity; the association between the functional connectivity within higher-order brain region (or within lower-order; or between higher and lower-order regions) and CSF Aβ42 was quantified across the early Aβ accumulator (CSF+/PET-) subjects.
